# Facilitating Sodium‐Ion Diffusion in Fe‐Doped Co_3_O_4_ for High‐Rate Performance

**DOI:** 10.1002/smll.202412449

**Published:** 2025-02-28

**Authors:** Yonghuan Fu, Guowei Sun, Rene Lucka, Qijun Song, Franz Renz, Huaping Zhao, Zhijie Wang, Yong Lei

**Affiliations:** ^1^ Fachgebiet Angewandte Nanophysik Institut für Physik & IMN MacroNano Technische Universität Ilmenau 98693 Ilmenau Germany; ^2^ School of Chemical and Material Engineering Jiangnan University WuXi 214122 China; ^3^ Institut für Anorganische Chemie Leibniz Universität Hannover 30167 Hannover Germany; ^4^ Laboratory of Solid-State Optoelectronics Information Technology Beijing Key Laboratory of Low Dimensional Semiconductor Materials and Devices, Institute of Semiconductors Chinese Academy of Sciences Beijing 100083 China; ^5^ Center of Materials Science and Optoelectronics Engineering University of Chinese Academy of Sciences Beijing 100049 China

**Keywords:** electronic conductivity, high‐rate performance, iron‐doped Co_3_O_4,_ sodium‐ion diffusion, transport spacing

## Abstract

Due to its high theoretical capacity, cobalt oxide (Co_3_O_4_) has attracted attention to sodium‐ion battery (SIB) anodes. However, its low conductivity and poor rate performance have limited its practical application. This work proposes a co‐precipitation doping strategy to synthesize iron‐doped Co_3_O_4_ nanoparticles (Fe_x_Co_3‐x_O_4_ NPs). Both experimental and theoretical results confirm that iron (Fe) doping at octahedral sites within spinel structures is a critical factor in enhancing rate performance. The decreased bandgap and enlarged ion transport spacing originate in Fe doping. This effectively facilitates the electron and Na‐ion (Na^+^) transport during discharge/charge processes, delivering an impressive rate capability of 402.9 mAh g^−^¹ at 3 A g^−^¹. The Fe_x_Co_3‐x_O_4_ NPs demonstrate remarkable cycling stability. They maintain a high specific capacity of 786.2 mAh g^−^¹ even after 500 cycles at 0.5 A g^−^¹, with no noticeable capacity fading. When assembled into a Na‐ion full cell, a remarkable discharge capacity of 105 mAh g^−1^ with stable cycling performance is attained. This work provides valuable insights into the functional design of high‐rate electrodes, offering a promising approach to addressing the critical challenges faced by sodium anodes.

## Graphical abstract

The limited rate performance of cobalt oxide (Co_3_O_4_) materials restricts their applicability in the rapid charge–discharge process of sodium‐ion batteries (SIBs). Iron (Fe) lattice doping can facilitate sodium‐ion diffusion by narrowing the bandgap and modulating the Co─O bond length. This study provides valuable insights into the functional design of high‐rate anode electrodes.

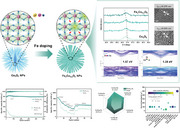

## 1 Introduction

The ever‐increasing global energy demand has intensified the search for sustainable and efficient energy storage systems. Sodium‐ion batteries (SIBs) have gained significant attention among promising candidates due to their abundant resources, safety, and environmental friendliness.^[^ ^1^ ^]^ However, SIBs suffer from low capacity and poor rate performance due to the large ionic radius of Na‐ion (Na^+^) in practical applications.^[^ ^2^, ^3^, ^4^ ^]^ Various materials have been developed as anodes to enhance the electrochemical performance of SIBs during charge and discharge processes, including carbon‐based materials, transition metal sulfides (TMSs), alloying materials, and transition metal oxides (TMOs).^[^ ^1^ ^]^ Carbon‐based materials have limited capacities due to their inadequate Na^+^ storage capability.^[^ ^5^, ^6^, ^7^, ^8^ ^]^ Transition metal sulfides (TMSs) and alloying materials have been widely explored as anode candidates for sodium‐ion batteries (SIBs) due to their high theoretical capacities and favorable reaction kinetics.^[^ ^9^, ^10^, ^11^ ^]^ However, TMSs often suffer from poor cycling stability caused by severe volume expansion and structural degradation during sodiation/desodiation. Additionally, their inherent low electrical conductivity hinders rate performance. On the other hand, alloying materials (such as Sn, Sb, and Bi‐based compounds) exhibit high capacities through alloying‐dealloying reactions but experience substantial volume changes, leading to rapid capacity fading and poor long‐term durability.^[^ ^12^, ^13^, ^14^ ^]^ In comparison, transition metal oxides (TMOs) offer a more balanced electrochemical performance, featuring moderate capacity, improved structural stability, and enhanced cycling lifespan.^[^ ^1^, ^15^, ^16^ ^]^ Their relatively stable crystal structures mitigate severe volume expansion, and their diverse redox chemistry contributes to better electrochemical reversibility. Therefore, TMOs emerge as promising alternatives with superior overall performance compared to TMSs and alloying materials for high‐performance SIB anodes.

Cobalt oxide (Co_3_O_4_) has emerged as a potential anode material for SIBs due to its high theoretical capacity and behavior as a p‐type semiconductor with favorable electronic properties.^[^ ^17^, ^18^ ^]^ The intrinsic low electrical conductivity of Co_3_O_4_ has been a critical factor limiting its rate capability as an anode material. Furthermore, the sodium‐ion diffusion within the bulk structure is constrained by a singular diffusion path, which hinders efficient Na⁺ transport.^[^ ^1^, ^4^, ^19^ ^]^ Consequently, the Co_3_O_4_ electrode suffers from poor rate performance, with the impact of ion diffusion playing a more critical role than the associated volume expansion. Therefore, developing high‐performance anode materials remains a critical challenge, particularly in achieving high‐rate performance, which is essential for fast charging and high‐power applications. Improving the anode material's ionic and electronic conductivity is the key to enhancing rate performance.

Constructing porous structures is an innovative way to develop high‐rate performance anode materials to improve sodium storage performance in metal‐oxides‐based anodes.^[^ ^20^, ^21^ ^]^ However, the porous structure alone does not effectively address the inherently low conductivity of metal oxides or sufficiently mitigate the constrained diffusion pathways. These limitations can lead to the accumulation of inactive Na^+^ within the internal active material during repeated Na⁺ insertion and extraction cycles, resulting in “dead sodium”.^[^ ^22^ ^]^ Another effective method is constructing rich intrinsic conductivity cobalt oxide with iron lattice doping.^[^ ^18^, ^23^ ^]^ The improvement in electrical conductivity reduces impedance in the electron transport pathway, facilitating a more uniform and rapid electrochemical reaction. Notably, uniform reaction kinetics help mitigate the destructive effects of volume expansion, thereby reducing particle pulverization and material detachment.^[^ ^10^ ^]^ In addition, varying lattice components can modulate the lengthening of metal‐O bonds and the expansion of ion transport channels, leading to enhanced ionic kinetics.^[^ ^24^, ^25^ ^]^

Although constructing material with iron lattice doping has been shown to improve sodium storage properties, the anode capacity at the high‐rate performance of the formed anodes seems unsatisfactory.^[^ ^26^, ^27^, ^28^ ^]^ In addition, the functional mechanism of iron lattice doping on metal oxide is still being determined. Therefore, to understand electrochemical reactions at the atomic level, it is necessary to study the effect of iron lattice doping on sodium storage properties using a straightforward method.

In this work, we demonstrate an urchin‐like hollow‐structured electrode of iron lattice doping cobalt oxide nanoparticles (Fe_x_Co_3‐x_O_4_ NPs) for SIBs, delivering a reversible capacity of 402.9 mAh g^−1^ at 3 A g^−1^ and maintaining a high specific capacity of 786.2 mAh g^−^¹ even after 500 cycles at 0.5 A g^−^¹, which makes this work the best performance of capacity/cycles ever reported so far. Fe_x_Co_3‐x_O_4_ NPs show the unique high‐rate performance of sodium ion storage. Electrochemical investigations and density functional theory (DFT) calculations, which have not been reported previously in cobalt oxide anodes, elucidated the mechanism and importance of iron lattice doping, which can improve the Na^+^ diffusion coefficient and increase the intrinsic conductivity. This work presents a general approach for preparing high‐rate capacity electrode materials and also clearly explains the functional design of high‐rate electrodes.

## 2 Results and Discussion

### 2.1 Structure and Morphology

**Figure 1:**
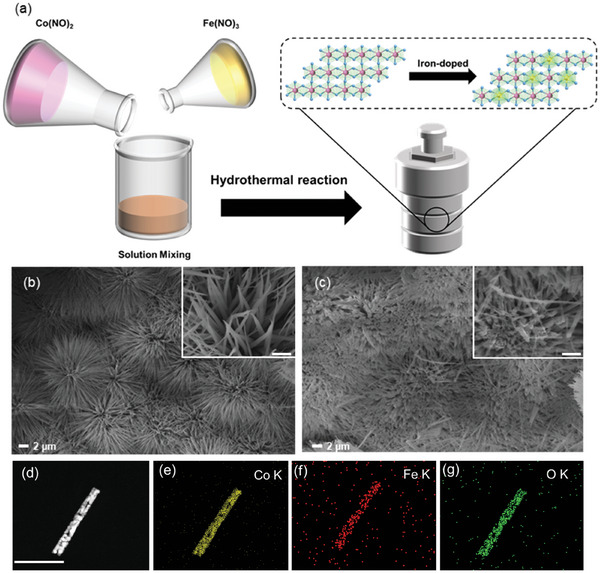
a) Schematic illustration of the preparation of Fe_x_Co_3‐x_O_4_ NPs. SEM images of b) Co_3_O_4_ NPs (inset in b is a SEM image of nanoparticles with high magnification, scale bar is 200 nm), c) Fe_x_Co_3‐x_O_4_ NPs (inset in b is a SEM image of nanowires with high magnification, scale bar is 20 nm). d) HAADF‐STEM image and elemental mapping analysis of Fe_x_Co_3‐x_O_4_ NPs; e) Co, f) Fe, and g) O elements, scale bar is 0.5 µm.

**Figure 2:**
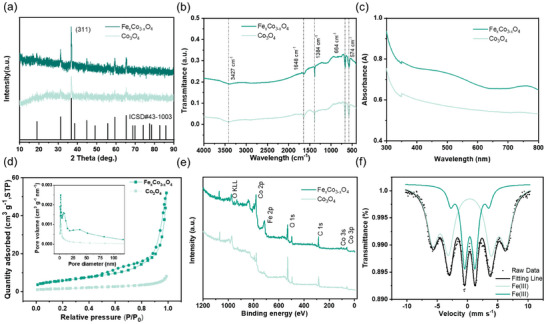
Structure characterizations of Fe_x_Co_3‐x_O_4_ NPs sample by spectroscopes. a) XRD pattern; b) FTIR spectrum; c) UV–vis spectrum; d) Nitrogen adsorption/desorption isotherms; e) XPS survey spectrum and e) Mössbauer spectra of Fe_x_Co_3‐x_O_4_ NPs at room temperature.

The urchin‐like Fe_x_Co_3‐x_O_4_ NPs were fabricated by the co‐precipitation doping synthesis method. **Figure** **1** shows the scanning electron microscopy (SEM) and high‐resolution transmission electron microscopy (HRTEM) images of the Fe_x_Co_3‐x_O_4_ NPs sample prepared by the hydrothermal reaction of Fe(NO_3_)_3_·9H_2_O, Co(NO_3_)_2_·6H_2_O, Ammonium fluoride (NH_4_F) and Urea in deionized (DI) water. The urchin‐like Fe_x_Co_3‐x_O_4_ NPs sample with iron is a lot of hollow microspheres with diameters of 1–2 µm and high surfaces (Figure 1c). The surface nanorods with size of 20–80 nm were grown, and subsequently formed into micron‐sized urchin‐like Fe_x_Co_3‐x_O_4_ NPs with the BET surfaces area ≈20.99 m^2^ g^−1^ (**Figure** **2**d). The characteristic of Fe_x_Co_3‐x_O_4_ NPs was further characterized with the HRTEM and high‐angle annular dark‐field scanning transmission electron microscope (HAADF‐STEM), as shown in Figure S1 (Supporting Information). Clearly, the well‐defined lattice interlayer spacing with 0.275 nm was observed, which is larger than the typical 0.243 nm spacing associated with the (311) plane of the cubic Co_3_O_4_ phase, which can be attributed to Fe lattice doping. The incorporation of Fe increases bond lengths within the crystal lattice, which in turn leads to an expansion in ion (Na^+^) transport channels. Meanwhile, the high‐resolution HAADF‐STEM image corresponding elemental energy‐dispersive X‐ray spectroscopy (EDS) mapping (Figure 1d–g) demonstrated that Co, Fe, and O were uniformly dispersed throughout the nanoparticles, suggesting that Fe were introduced during the hydrothermal process. Additionally, we obtained the atomic ratio of Co/Fe around 11:1 by the inductively coupled plasma‐optical emission spectrometer (ICP‐OES) technique, as shown in Table S3 (Supporting Information), further confirming the content of iron element. As a result, Fe_x_Co_3‐x_O_4_ NPs have been successfully obtained by the co‐precipitation doping synthesis method.

We also note that the Fe_x_Co_3‐x_O_4_ NPs and Co_3_O_4_ nanoparticles (Co_3_O_4_ NPs) samples present the same crystal structure, as evidenced by the corresponding XRD patterns in Figure 2a and Figure S2 (Supporting Information). The X‐ray diffraction (XRD) technique to analyze the crystallographic structure of Fe_x_Co_3‐x_O_4_ NPs and Co_3_O_4_ NPs. There are four prominent peaks located at 2θ = 31.2°, 36.8°, 59.3°, and 65.2°, as shown Figure 2a, which can be indexed to (220), (311), (511), and (440) diffractions of cubic Co_3_O_4_ phase (PDF # 43–1003), respectively. The lattice parameters of Fe_x_Co_3‐x_O_4_ NPs obtained through Rietveld refinement of XRD data using the FullProf Suite (version 5.10) are listed in Table S2 (Supporting Information). The lattice parameters increase with increasing iron lattice doping, which is consistent with the detailed XRD (Figure S2, Supporting Information). The lattice parameters further indicate that iron lattice doping increases the metal‐oxygen bond length. And the average crystallite size of Co_3_O_4_ calculated using Scherrer's formula is listed in Table S2 (Supporting Information). The decrease in average crystallite size with increasing doping suggests that doping may reduce average crystallite size and increase specific surface area. This result is consistent with the results of Brunauer–Emmett–Teller (BET) surface area analysis. The values of the lattice distortion are calculated in Table S2 (Supporting Information) by the Williamson–Hall formula.^[^ ^29^, ^30^, ^31^ ^]^ The lattice distortion values (**ε_hkl_**) of the Co_3_O_4_ and Fe_x_Co_3‐x_O_4_ NPs samples are estimated to be 2.39 × 10^−2^ and 2.76 × 10^−2^, respectively. It was found that Fe_x_Co_3‐x_O_4_ NPs exhibited higher lattice distortion values than that of Co_3_O_4_ because of iron element incorporation into the Co_3_O_4_ lattice. As Fe doping increases, the films’ surface roughness decreases, likely due to the change in lattice size shown in Table S2 (Supporting Information).^[^ ^32^ ^]^ This occurs due to iron integrating into the host cobalt oxide lattice, which is known to ensure a significant uniform electrochemical surface reaction.^[^ ^33^ ^]^ Meanwhile, iron‐induced dopant sites can be used as active sites to adsorb Na^+^ ions.^[^ ^34^, ^35^ ^]^ Therefore, incorporating iron ions into the cobalt oxide lattice decreases crystallite size and increases lattice parameters. Additionally, the formation of subtle lattice distortions at the iron‐doped sites contributes to a more uniform electrochemical surface reaction. The Fourier transform infrared (FTIR) spectra of Co_3_O_4_ NPs and Fe_x_Co_3‐x_O_4_ NPs are shown in Figure 2b. The peak at roughly 3427 cm^−1^ corresponds to the O─H stretching vibration of water molecules, while the smaller peak at ≈1648 cm^−1^ may be caused by the O─H stretching and bending modes of water molecules.^[^ ^33^ ^]^ The intensity of the OH stretching vibration absorption peak is reduced with adding iron ions, to Co_3_O_4_ NPs. Additionally, the peak roughly between 1384 cm^−1^ matches the O─H─Co stretching vibration. Finally, the characteristic peaks at 664 and 574 cm^−1^ are connected to the stretching vibrations of the metal–oxygen (M–O) bond, which supports the spinel structure of Co_3_O_4_ NPs. Due to the addition of iron, the band shifted slightly toward the higher wavenumber region.^[^ ^33^, ^36^ ^]^ Moreover, the FTIR spectra investigation are well matching with XRD data.

The UV–vis spectra (UV–vis) of Fe_x_Co_3‐x_O_4_ NPs and Co_3_O_4_ NPs samples are shown in Figure 2c. The absorption spectrum is the presence of two pronounced absorption edges in the visible region in UV–vis spectra, which are ascribed to the ligand‐to‐metal charge transmission result of (E_g2_, O^2−^ → Co^2+^) and (E_g1_, O^2−^ → Co^3+^) in Co_3_O_4_. The optical bandgap of Co_3_O_4_ NPs and Fe_x_Co_3‐x_O_4_ NPs sample were calculated using Tauc relation from Equation S1 (Supporting Information).^[^ ^36^ ^]^ Tauc plot of Co_3_O_4_, and Fe_x_Co_3‐x_O_4_ NPs were calculated by extrapolating the linear part of these plots of (αhν)^2^ axis to (hν) axis (Figure S3, Supporting Information).^[^ ^33^ ^]^ The bandgap values are decreased due to the addition Fe^3+^ ions (Table S1, Supporting Information). The maximum bandgap (E_g2_) for Co_3_O_4_ NPs is assigned to the valence to conduction band excitation. In contrast, the minimum bandgap (E_g1_) is assigned to the O^2−^ → Co^3+^ charge transfer at Fe_x_Co_3‐x_O_4_ NPs where Co^3+^ ions are located below the conduction band; due to this impurity energy levels are created in the bandgap region. In contrast, Fe contributes to hole generation and increases its role with the number of charge carriers (holes) that contribute to the conductivity of Fe_x_Co_3‐x_O_4_ NPs. DFT calculations will further validate this result (Figure 5c,d). The four‐point probe conductivity measurements presented in Table S1 (Supporting Information) reveal a clear and systematic trend: the electrical conductivity of Fe_x_Co_3‐x_O_4_ NPs improves progressively with increasing Fe doping levels. This enhancement in conductivity can be attributed to Fe‐doped electronic structure modification, facilitating charge carrier mobility through the lattice.^[^ ^37^, ^38^, ^39^, ^40^ ^]^ Notably, the 6% Fe‐doped sample achieves the highest electrical conductivity among the tested compositions, with a value of 11.64 × 10^−3^ S cm^−1^, underscoring the optimal balance between doping concentration and electrical conduction.

The N_2_ adsorption/desorption was measured at 77 K with a relative pressure P/P_0_ ranging from 0.029 to 0.99. From these adsorption/desorption curves, BET‐specific surface areas and corresponding Barrett‐Joyner‐Halenda (BJH) pore sizes were calculated for Fe_x_Co_3‐x_O_4_ NPs, as shown in Figure 2d. These curves show the most significant number of pores distribution ≈5–15 nm for all Fe_x_Co_3‐x_O_4_ NPs. This pore size distribution range could help boost the diffusion kinetics inside the electrode material.^[^ ^34^ ^]^ Table S1 (Supporting Information) lists measured BJH pore radius, and BET surface area of Fe_x_Co_3‐x_O_4_ NPs samples. The reported higher surface area, in the range of 5.70–20.99 m^2^ g^−1^, of samples indicates that the hydrated electrolyte ions could have a high contact area at the electrolyte/electrode surface for the Faradaic redox reaction.^[^ ^11^ ^]^ The enhancement in electrochemistry activity may have been influenced by the expansion of active sites resulting from the increased surface area, as well as the improved conductivity due to the reduction in the bandgap.^[^ ^34^ ^]^

The electron interactions and the surface composition of the elements of Fe_x_Co_3‐x_O_4_ NPs were also revealed by X‐ray photoelectron spectroscopy (XPS) measurements (Figure 2e). As shown in Figure 2e, the full XPS survey of Fe_x_Co_3‐x_O_4_ NPs exhibits the existence of cobalt, iron, and oxygen elements. The contents of Co and Fe were 20.60% and 3.36%, respectively, calculated from the XPS data. Especially, there are Fe 2p signal peaks were observed in the full XPS survey of Fe_x_Co_3‐x_O_4_ NPs. The Co 2p spectrum of Fe_x_Co_3‐x_O_4_ NPs can be deconvoluted into four distinct peaks (Figure S4, Supporting Information), with the peaks at 779.7 and 794.8 eV corresponding to the Co^3+^ components of the 2p_3/2_ and 2p_1/2_ states, respectively.^[^ ^41^ ^]^ In addition, the peaks at 781.5 and 796.9 eV represent the binding energies of Co^2+^ for the 2p_3/2_ and 2p_1/2_ states. Compared to Co_3_O_4_ NPs, the Co 2p_3/2_ and 2p_1/2_ peaks in the XPS spectrum of Fe_x_Co_3‐x_O_4_ NPs shift toward higher binding energies, indicating changes in the electronic environment due to iron lattice doping. Notably, both Co^3+^ and Co^2+^ signals are present in Fe_x_Co_3‐x_O_4_, as shown in the XPS fine spectra (Figure S4, Supporting Information), along with clear satellite peaks. The observed decrease in the mole ratio of Co^3+^ relative to Co^2+^ confirms that Fe^3^⁺ substitution for Co^3+^ occurs at octahedral sites. This substitution reduces the proportion of Co^3+^, increasing the presence of Co^2+^. The core‐level O 2p spectrum of Fe_x_Co_3‐x_O_4_ NPs in Figure S5 (Supporting Information), the peaks located at 528.2, 530, and 532.1 eV can be ascribed to the metal‐oxide bonds. Among these the peak at 530.0 eV should be attributed to the lattice oxygen.^[^ ^42^ ^]^ The peaks at 532.1 eV are the surface oxygen and water molecules adsorbed on the surface element.^[^ ^21^, ^43^ ^]^ The high peak area content at 532.1 eV also confirmed to the rich porous structure of the Fe_x_Co_3‐x_O_4_ NPs. Compared with those of Co_3_O_4_ NPs, the O 1s in the XPS spectrum of Fe_x_Co_3‐x_O_4_ NPs positively shift ≈2.24 eV (Figure S5, Supporting Information). The replacement of Co^3+^ by Fe^3+^ is further supported by the shift in binding energy observed for the O 1s peak (Figure S5, Supporting Information). Due to the slightly lower electronegativity of Fe^3+^ (1.83) compared to Co^3+^ (1.88), the electron density around oxygen atoms decreases, leading to an increase in the binding energy of the O 1s peak, which consequently shifts to higher binding energies. Therefore, the above results confirm Fe in the Fe_x_Co_3‐x_O_4_ NPs, and the Fe^3+^ replaces part of Co^3+^ at octahedral sites in the Fe_x_Co_3‐x_O_4_ NPs. We further analyze the XPS spectra of Fe 2p as shown in Figure S6 (Supporting Information), the Fe 2p doublet peaks at 710.0, 711,8, and 713.9 eV are correlated with the Fe^3+^, respectively, which can be attributed to the metal‐oxide bonds of Fe_x_Co_3‐x_O_4_ NPs.^[^ ^44^, ^45^, ^46^ ^]^ The Mössbauer spectrum of Fe_x_Co_3‐x_O_4_ NPs reveals again that the doped iron exists exclusively in the Fe(III) oxidation state (Figure 2f). This result is consistent with the XPS data (Figure S6, Supporting Information). Both primary peaks, represented by the dark and light green lines, indicate Fe^3+^ ions in octahedral coordination. The dark green line is associated with Fe^3+^ ions having a higher hyperfine magnetic field (37.578 T), which is characteristic of the spinel structure in Co_3_O_4_.^[^ ^47^, ^48^ ^]^ While the light green line corresponds to Fe^3+^ ions with a slightly lower field (33.947 T). This may be due to the difference in the local environment due to lattice distortion caused by iron lattice doping.^[^ ^49^ ^]^ As shown in Table S4 (Supporting Information), the isomer shift (δ) of ^57^Fe at the octahedral site increases from 0.35 to 0.37 mm s^−1^. An increase in δ might reflect a shift in the local chemical environment due to bonding length changes.

### 2.2 Electrochemical Performance

**Figure 3:**
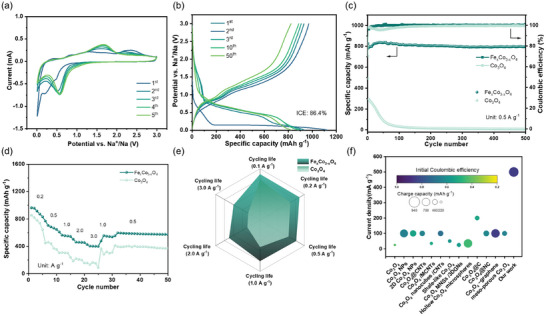
CV curves of Fe_x_Co_3‐x_O_4_ NPs electrode within a) 0.01–3 V at a scan rate of 0.1 mV s^−1^. Galvanostatic charge/discharge profiles of the Fe_x_Co_3‐x_O_4_ NPs electrode at a current density of 500 mA g^−1^ within b) 0.01–3 V. c) Cycling performance of Fe_x_Co_3‐x_O_4_ NPs and Co_3_O_4_ NPs electrode at 500 mA g^−1^ within 0.01–3 V. d) Rate performance of Fe_x_Co_3‐x_O_4_ NPs and Co_3_O_4_ NPs electrode. e) The cycling life of Fe_x_Co_3‐x_O_4_ NPs and Co_3_O_4_ NPs with different current densities, the capacity retention is <50%. f) Comparison of performance data with other anode materials for SIBs reported in recent literature.

The electrochemical performance of the Fe_x_Co_3‐x_O_4_ NPs electrode was evaluated by assembling half‐cell SIBs. As displayed in **Figure** **3**a, the initial five cyclic voltammetry (CV) curves of the Fe_x_Co_3‐x_O_4_ NPs electrode at a scan rate of 0.1 mV s^−1^ within the potential window of 0.01–3.0 V to reveal the sodiation/desodiation reaction mechanism. The shapes of the CV curves and current intensity are well maintained after the first cycle, demonstrating the excellent reversibility for sodiation/desodiation of the Fe_x_Co_3‐x_O_4_ NPs electrode in both voltage ranges. Based on the electrochemical performance and ex situ/in situ characterization analysis, the conversion reaction mechanism of Fe_x_Co_3‐x_O_4_ NPs with Na metal may occur in two steps, which can be expressed as following equations:

(1) $$Fe_{x}Co_{3-x}O_{4}+2Na^{+}+2e^{-}↔\;Na_{2}O+\mathrm{xFeO}+3-x\;\mathrm{CoO}$$

(2) $$3-x\;\mathrm{CoO}+2\left(3-x\right)\;Na^{+}+2\left(3-x\right)\;e^{-}↔\;3-x\;Na_{2}O+3-x\;\mathrm{Co}$$

(3) $$\mathrm{Or}x\mathrm{FeO}+2x\;Na^{+}+2x\;e^{-}↔\;x\;Na_{2}O+x\;\mathrm{Fe}$$

**Figure 4:**
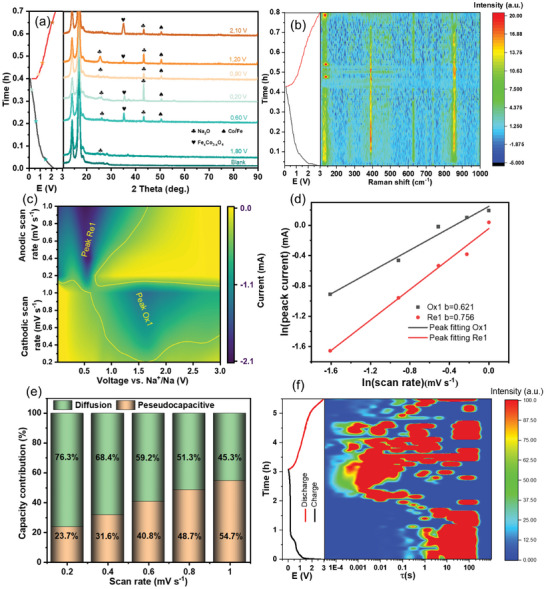
Reaction mechanism investigation of Fe_x_Co_3‐x_O_4_ NPs electrode for SIB a) ex situ XRD patterns at various potential states. b) in situ Raman spectra of Fe_x_Co_3‐x_O_4_ NPs. c) the contour of the Fe_x_Co_3‐x_O_4_ NPs electrode at different scan rates. d) ln(*i*)–ln(*v*) curves (b‐value determination). e) Contribution ratios of the surface process in the Fe_x_Co_3‐x_O_4_ NPs electrode at different scan rates. (f) DRT analysis based on the in situ GEIS of Fe_x_Co_3‐x_O_4_ NPs.

In the CV curves of the Fe_x_Co_3‐x_O_4_ NPs electrode in the potential window of 0.01‐3 V, there are several reduction peaks at 0.60 and 0.20 V in the first reduction scan, which correspond to the oxidation peak at ≈1.60 and 2.10 V, respectively.^[^ ^1^, ^50^, ^51^ ^]^ These redox peaks are attributed to the multiple‐step intercalation of Na^+^ into Fe_x_Co_3‐x_O_4_ NPs and subsequent conversion reactions. The corresponding phase transition reaction process can be observed in Figure S7 (Supporting Information). Interestingly, the discharge‐specific capacity of the Fe_x_Co_3‐x_O_4_ NPs electrode increases slowly to a very high capacity and then can remain stable after many cycles. As shown in Figure 3b, the charge curves of each cycle nearly overlap in the section of 0.01–0.60 V, but the curves gradually become a wider plateau at ≈0.60 and 1.20 V with the increase of cycle number, indicating at this voltage the main electrochemical conversion reactions are taking place. It is worth mentioning that the cobalt oxide (CoO) often undergoes the reversible conversion reaction between Co and Fe_x_Co_3‐x_O_4_, which can be confirmed from ex situ XRD (**Figure** **4**a) and in situ Raman (Figure 4b). Therefore, the high‐oxidation end product after recharge to 3.0 V indicates the enhanced kinetics of conversion reactions for the as‐prepared electrodes. The conversion reactions agreed well with the galvanostatic charge–discharge curves. Compared with the increasing cycle of discharge profiles, two sloping plateaus gradually emerge ≈1.60 and 0.80 V. Furthermore, the charge and discharge curves have the same shape in the range of 0.01–3 V, indicating that a strong reversibility of the conversion reaction. It is worth mentioning that a similar redox couple can be observed in the CV curves of the pure Co_3_O_4_ NPs electrode (Figure S8, Supporting Information), indicating this is a common electrochemical side reaction in Co‐based oxide.^[^ ^1^, ^16^ ^]^ The following chapter will provide an in‐depth analysis of the conversion reactions, utilizing both in situ Raman and ex situ XRD.

The Fe_x_Co_3‐x_O_4_ NPs show excellent cycle performance in Figure 3c. The typical charge/discharge curves of the Fe_x_Co_3‐x_O_4_ NPs electrode for the first cycles at 500 mA g^−1^ exhibited a high initial discharge/charge capacity of 949.6/820.8 mAh g^−1^ with a remarkable initial Coulombic efficiency (ICE) of 86.4% within the voltage range of 0.01–3 V. The irreversible capacity loss is due to the formation of a solid electrolyte interphase (SEI) layer. The Fe_x_Co_3‐x_O_4_ NPs electrode exhibits outstanding cycling stability, maintaining a high specific capacity of 786.2 mAh g−¹ even after 500 cycles at 500 mA g^−^¹. The coulombic efficiency of the Fe_x_Co_3‐x_O_4_ NPs electrode is around 100% (500 mA g^−1^) at the fewest charge/discharge cycles, thus reflecting the rapid construction of the stability SEI film. Noticeably, the reversible capacity of Fe_x_Co_3‐x_O_4_ NPs is higher than that of the theoretical capacity of 890 mAh g^−1^ with 6 Na^+^ storing.^[^ ^22^ ^]^ The excess reversible capacity might be ascribed to synergistic effects with the supper P. The reversible capacity contribution of the conductive additive supper P is 164.8 mAh g^−1^ within 0.01‐3 V.^[^ ^52^ ^]^ To further investigate the rate performance of Fe_x_Co_3‐x_O_4_ NPs, they were conducted at various current densities. The corresponding 5th discharge capacities of the electrode were 916.2, 667.8, 552.3, 458.6, and 402.9 mAh g^−1^, with the current densities gradually increasing to 0.2, 0.5, 1, 2, and 3 A g^−1^ within the voltage range of 0.01–3 V, respectively (Figure 3d). It is worth mentioning that an unexpected specific capacity of 402.9 mAh g^−1^ can be achieved at a high current density of 3.0 A g^−1^. Fe_x_Co_3‐x_O_4_ NPs exhibited a stable performance for 150 cycles at a current density of 3.0 A g^−1^ as shown in Figure S20 (Supporting Information). It is especially noteworthy that the high‐rate response at 3.0 A g^−1^ means an ultrafast charge/discharge time of <15 min, highlighting the high‐rate application of the Fe_x_Co_3‐x_O_4_ NPs electrode in SIBs. Furthermore, the current density returned to 500 mA g^−1^, and the capacity of Fe_x_Co_3‐x_O_4_ NPs can also reach 592.8 mAh g^−1^, which demonstrated excellent reversibility and superior stability of the Fe_x_Co_3‐x_O_4_ NPs anode electrode toward SIBs. Figure 3e presents the number of cycles recorded at different charge/discharge currents. A representative cycling curve at a current density of 2.0 A g^−^¹ is presented in Figure S21 (Supporting Information). As shown in Figure 3e, the Fe_x_Co_3‐x_O_4_ NPs electrode demonstrates a remarkable cycle life, maintaining its capacity over an extended number of cycles, thereby indicating its potential for long‐term stability and reliability in battery applications. Compared with the cobalt oxide anode reported in recent years (Table S8, Supporting Information), which includes the capacities, rate capabilities, ICE, cycling performance, and voltage window, it is clear that the rate performance of the Fe_x_Co_3‐x_O_4_ NPs electrode is the best among the reported cobalt oxide anode materials in SIBs (Figure 3f).

### 2.3 Kinetics and Reaction Mechanism

We further explored the origin of the superior rate performance in the Fe_x_Co_3‐x_O_4_ NPs electrode and investigated the kinetics characterization by electrochemical characterization techniques (Figure 4). To reveal the Na^+^ storage mechanism and the formation of excellent rate performance of Fe_x_Co_3‐x_O_4_, the post‐treatment electrode was investigated based on ex situ XRD, in situ Raman, and in situ galvanostatic electrochemical impedance spectra (in situ GEIS) at various depths of the second discharge/charge. The selected XRD patterns of the Fe_x_Co_3‐x_O_4_ NPs at various potential states (including open circuit voltage [OCV]) were performed during the first cycle at 100 mA g^−1^, as depicted in Figure 4a. In the ex situ XRD analysis, characteristic XRD peaks for the Na_2_O crystal phase were observed at voltages of 1.80, 0.60, 0.20, and 1.20 V. Similarly, distinct signal peaks corresponding to Co were detected at 0.60, 0.20, 0.80, 1.20, and 2.10 V. This is consistent with the results of the reaction equations mentioned in Chapter II.

Cyclic Voltammetry (CV) measurements of Fe_x_Co_3‐x_O_4_ NPs electrodes are performed at scan rates from 0.2 to 1 mV s^−1^. As shown in Figure 4c and Figure S9a (Supporting Information), by converting the CV curve into a contour plot, the position of the oxidation and reduction peaks can be clearly. The contour plot was derived from the CV curve at different scanning rates provide a more detailed visualization of peak position shifts (Figure S15, Supporting Information). Interestingly, cathodic and anodic scans were able to observe the oxidation peak at 1.75 V and the reduction peak at 0.75 V respectively, which agrees well with the Na^+^ conversion reaction of the galvanostatic charge/discharge profile in Figure 3b, with an obvious distinct plateau area. As we know, according to the relationship between peak currents (*i*) and scan rates (*v*) (Equations S1 and S2, Supporting Information), we can obtain the *b* value.^[^ ^1^, ^53^ ^]^ In general, values of *b* close to 0.5 indicate a diffusion‐controlled process, whereas values of *b* approaching 1.0 represent a capacitive process. As depicted in Figure 4d, both display the fitted slope *b*‐values of ≈0.5 for Fe_x_Co_3‐x_O_4_ NPs electrodes, suggesting that the electrochemical process is dominated by a combination of surfaced‐controlled pseudocapacitive and diffusion‐controlled behavior for Na‐ion storage. For anode material surface control, pseudocapacitive can provide high capacity. Moreover, the quantification of the capacity contribution ratio can be further calculated from Equation S3 (Supporting Information). As results, the capacitive contribution ratio of the Fe_x_Co_3‐x_O_4_ NPs electrode gradually increases from 26% to 57% (Figure 4e) for an electrode. The detailed of the kinetic analysis are displayed in Table S5 (Supporting Information). The Fe_x_Co_3‐x_O_4_ NPs electrode shows a higher diffusion contribution ratio at the same scan rate than the Co_3_O_4_ electrode (Figures S9b and S15b, Supporting Information) in the sodiation process, which suggests that sodium ion diffusion plays a critical role in determining the electrochemical properties of the Fe_x_Co_3‐x_O_4_ NPs. At a scan rate of 0.6 mV s^−1^, the contour of the pseudocapacitance contribution is closely aligned with the CV curve in Figure S10 (Supporting Information), confirming the validity of the pseudocapacitance fitting. The kinetics of Na^+^ diffusion coefficient (D_Na_^+^) of the Fe_x_Co_3‐x_O_4_ NPs electrode is also investigated by CV at various scan rates (Figure S15a, Supporting Information) and calculated using the Randles–Sevcik equation (Equation S4, Supporting Information). According to the linear fitting results of I_p_ and v^1/2^ (Figure S11, Supporting Information), the D_Na_^+^ of the Fe_x_Co_3‐x_O_4_ NPs electrode is estimated to be 7.68 × 10^−12^ cm^2^ s^−1^, which is two orders of magnitude greater than that of Co_3_O_4_ (6.59 × 10^−14^ cm^2^ s^−1^) electrodes. This result is highly consistent with the sodium ion diffusion coefficients obtained from the GITT test (Figure S12, Supporting Information), demonstrating comparable magnitudes and reinforcing the reliability of the measurements across both methods. Note that the electrochemical performance of anode materials in SIBs depended on the competition between adsorption and diffusion of ions on the host. Therefore, the high Na^+^ diffusion coefficient of the Fe_x_Co_3‐x_O_4_ NPs electrode will boost the electrochemical performance for sodium ion storage.

Electrochemical impedance spectroscopy (EIS, Figure S13, Supporting Information) was performed to better understand the effect of Fe doping on its kinetic properties and reveal the reaction resistance changes. As can be seen in Figure S13 and Table S6 (Supporting Information), the charge transfer resistance (R_ct_) of the Fe_x_Co_3‐x_O_4_ NPs electrode were decreased and then increased. This is consistent with the results of in situ GEIS, where the reaction process is dominated by charge diffusion. It is shown that the electron transfer kinetics can be effectively improved with the iron lattice doping for the electrode material. When the Fe_x_Co_3‐x_O_4_ NPs electrode material is discharged, the R_ct_ value of the Fe_x_Co_3‐x_O_4_ NPs electrode at 0.60 V is lower (67.05 Ω), showing the faster charge‐transfer for Na^+^ with the increase of capacity. *R*_ct_ decreased steadily due to the gradual decomposition of the SEI layer.^[^ ^54^ ^]^ This result can be confirmed from the galvanostatic intermittent titration technique (GITT) test curve (Figure S12, Supporting Information). The Na^+^ diffusion coefficient has a tendency to increase at 0.60 V. When the Fe_x_Co_3‐x_O_4_ NPs electrode is charged, the R_ct_ value of Fe_x_Co_3‐x_O_4_ NPs electrode at 1.20 V is lower (29.84 Ω), The higher Na^+^ diffusion coefficient can be observed clearly. The in situ GEIS was conducted to explore the internal state associated with different potential levels, which is using infinite parallel resistance (R) and capacitance (C) to fit a complex system. Distribution of relaxation time (DRT) analysis was obtained based on in situ GEIS data. The τ value in DRT corresponds to electronic conductivity (10^−6^–10^−5^), particle and current collector contact (10^−5^–10^−4^), cathode electrolyte interface (10^−4^∼0.01), charge transfer in electrodes (0.01–10), and solid‐state diffusion in electrodes (10–300), respectively.^[^ ^55^, ^56^ ^]^ The DRT results of half batteries were decoupled by excluding the effect of the Na metal anode. The electronic resistance of the Fe_x_Co_3‐x_O_4_ NPs sample is consistently lower than that of the Co_3_O_4_ NPs sample throughout the entire charge–discharge process (Figure S14, Supporting Information), which is consistent with the above analysis. This finding highlights the material's voltage‐dependent resistance evolution and provides insight into the diffusion coefficient occurring during the electrochemical processes.

To further study the cycle stability of the Fe_x_Co_3‐x_O_4_ NPs electrode, SEM and TEM were performed on the cycled electrodes (over 200 cycles) in the electrochemical process. A uniform SEI film was generated on the surface of Fe_x_Co_3‐x_O_4_ NPs, and the sea urchin‐like morphology remained intact, as shown in Figure S16 (Supporting Information). The main component of the SEI layer is Na_2_O can be obtained from HRTEM (Figure S17, Supporting Information) and elemental mapping (Figure S19, Supporting Information).^[^ ^1^, ^39^, ^54^ ^]^ Conversely, the Co_3_O_4_ NPs electrode was encapsulated by a thick SEI film (Figure S16, Supporting Information), and a clear morphology of pulverized particles was observed. Additionally, TEM images of these electrodes after the 200th charge, Figure S17 (Fe_x_Co_3‐x_O_4_ NPs, Supporting Information) and Figure S18 (Co_3_O_4_ NPs, Supporting Information), reveal that a uniform SEI layer (2–3 nm) was generated on the surface of the Fe_x_Co_3‐x_O_4_ NPs electrode and needle‐like morphology is maintained after cycling (Figure S17c–d, Supporting Information), consistent with elemental mapping observation (Figure S19a, Supporting Information). Based on previous literature reports. Cobalt‐based oxides with needle‐like morphology have good cycling stability.^[^ ^57^ ^]^ The morphological evolution of Co_3_O_4`_ electrodes before and after cycling is presented in Figure S18 (Supporting Information). After cycling, the initially pristine Co_3_O_4_ NPs undergo significant pulverization, resulting in the formation of smaller fragmented particles dispersed around larger agglomerates. The elemental mapping of Co_3_O_4_ NPs after cycling was displayed in Figure S19b (Supporting Information). This structural degradation highlights the substantial mechanical stress and volume changes experienced during repeated charge/discharge cycles. In short, the high‐rate performance mainly stems from multiple factors: i) the conversion reaction and surface pseudocapacitance provide high capacity; ii) the lower electronic resistance and higher diffusion coefficient during discharge/charge processes help to form the SEI film uniformly; iii) Enhancing of electrical conductivity reduces internal stress and partially mitigates volume expansion, contributing to improved structural stability during cycling.

### 2.4 Theoretical Calculations on the Effect of Iron Lattice Doping on Conductivity

**Figure 5:**
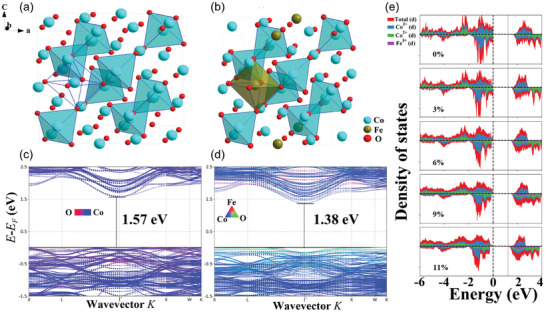
The corresponding crystallographic structures of a) Co_3_O_4_ NPs and b) Fe_x_Co_3‐x_O_4_ NPs unit cells, with one of the Co^3+^ polyhedral depicted is transparent. c) Band structure of Co_3_O_4_ NPs and d) Fe_x_Co_3‐x_O_4_ NPs unit cells, with spin‐up (solid lines) and spin‐down (dashed lines) components. Iron atoms are randomly doped at the 16d Wyckoff sites. e) Projected density of states (PDOS) for the d orbitals in different doping content of Fe_x_Co_3‐x_O_4_.

The electron configurations of the 3d orbitals of cobalt and iron prominently dominate the conductivity of Fe_x_Co_3‐x_O_4_ NPs. We elucidated the underlying mechanisms that enhance the material's conductivity by analyzing the projected density of states (PDOS) of the cobalt and iron atoms within distinguished valance states. The bandgap values of the two models were calculated by constructing a unit of Co_24_O_32_ (**Figure** **5**a) and replacing some iron atoms to form a new crystal unit (Figure 5b). The bandgaps of Fe_2_Co_22_O_32_ (6%) decline from 1.57 to 1.38 eV with the addition of two iron atoms in the unit cell (Figure 5c,d). Due to the presence of iron, the bandgap values for both the total and spin‐up states narrow from 1.57 and 1.75 eV to 1.29 and 1.53 eV, respectively (Table S7, Supporting Information). When iron atoms are doped, the spin‐down electrons at the conduction band bottom shift slightly toward the Fermi energy level. As shown in Figure 5e, the partial orbitals of tetrahedral coordinated divalent cobalt decrease in energy from ≈3.5 eV to ≈2 eV, which increases the density of states in the conduction band. This, in turn, allows more charge carriers to be available for conduction and enhances the material's electrical conductivity. A detailed simulation can be obtained in Table S7 (Supporting Information). Notably, when iron lattice doping between Co_3_O_4_, the bandgap near the Fermi level is significantly reshaped, resulting in a higher density of electronic state (Figure 5e). Significantly, previous investigation results on iron lattice doping in Co_3_O_4_ will be presented in **Table** **1**, as a supplementary reference for our findings. Bhisikar et al. investigated the distribution of iron composition at different iron lattice doping levels.^[^ ^58^ ^]^ By summarizing the data, it was found that the doping concentration exceeds 8% (Co_2.6_Fe_0.34_O_4.64_), leading to Fe^2+^ substitution of Co^2+^ in the tetrahedral sites. This observation may account for the lower conductivity of 9% Fe doping compared to 6% Fe doping (Table S1, Supporting Information). As illustrated in Table S2 (Supporting Information), the incorporation of iron leads to the elongation of the Fe─O bond lengths within the octahedrally coordinated iron and cobalt atoms. This observation is consistent with experimental findings (Figure S2, Supporting Information).^[^ ^35^, ^59^ ^]^ The doped iron atoms at the 8*a* site contribute to an increase in the volume of their tetrahedral coordination while simultaneously reducing the Co/Fe 16*d*‐O bond lengths. This finding further confirms the enhanced electronic conductivity of Fe_x_Co_3‐x_O_4_ NPs compared to Co_3_O_4_ NPs. Iron lattice doping can reduce the bandgap between the conduction band and the valence band, enhancing electrical conductivity. This indicates that the presence of iron lattice doping sites is more favorable for the high‐rate performance of sodium ions.

**Table 1:** Experimental lattice parameters, bond lengths of pristine Co_3_O_4_ and its variants with doped iron atoms. (*r.t*.: room temperature).

Database(ICSD)	Co_3_O_4_ *r.t*.^[^ ^60^ ^]^	Co_2.95_Fe_0.05_O_3.947_ 300 *K* ^[^ ^58^ ^]^	Co_2.75_Fe_0.25_O_4.049_ 300 *K*	Co_2.6_Fe_0.34_O_4.64_ 300 *K*	Co_2.452_Fe_0.34_O_4_ 300 *K*
Cell parameter: a (Å)	8.08	8.11	8,17	8.17	8.17
Co 8*a*‐ O(Å)	1.94	1.91	1.91	1.95 (Fe^2+^ doping)	1.94 (Fe^2+^ doping)
Co/Fe 16*d* – O(Å)	1.92	1.94	1.96	1.95	1.94

### 2.5 Fe_x_Co_3‐x_O_4_ Versus PB Full Cell Study

**Figure 6:**
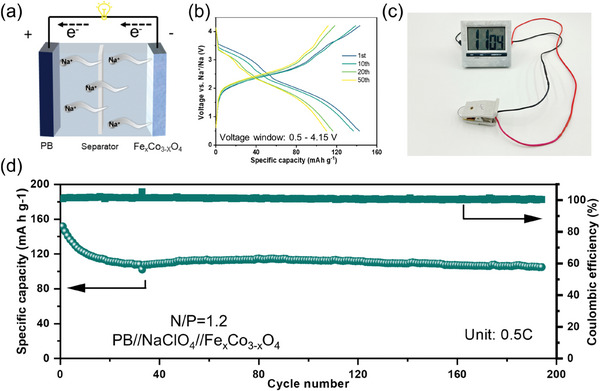
Electrochemical evaluations of the PB//NaClO_4_//Fe_x_Co_3‐x_O_4_ full cell. a) Schematic of the working process of SIBs, b) Detailed charge/discharge curves (0.5 C, 1 C = 170 mA g^−1^), c) Application demonstration powered by the full cell, d) Cycling stability (0.5 C).

The as‐prepared Fe_x_Co_3‐x_O_4_ anode material was charged/discharged at a current density of 0.05 A  g^−1^ for five cycles to form a stable SEI layer. The schematic illustration of the full cell is shown in **Figure** **6**a. For the experiment, the Prussian blue (PB) cathode was prepared as discussed in the Experimental Section by following the reported procedure.^[^ ^61^, ^62^ ^]^ The constructed full cell was cycled in the 0.5–4.15 V voltage range at a current rate of 0.5 C (1 C = 170 mA g^−1^). Figure 6b shows the charge–discharge profile of the full cells for the 1st, 10th, 20th, and 50th cycles, which shows similar charge–discharge reactions during each cycle. To test the feasibility of full batteries for real‐world applications, we turned on the timer using a single coin cell, as shown in Figure 6c, which confirms the present technology for further developments in the field of transition metal oxides for future battery applications. Figure 6d shows the cycling behavior of the full cell up to ≈200 cycles, a discharge capacity of ≈110  mAh g^−1^ was achieved after the 150th cycle by retaining ≈93% of the cell's initial capacity. Further, a considerable energy density of ≈255 Wh kg^−1^ at ≈3.65 V region confirms the full cell's efficient energy conversion.

## 3 Conclusion

Fe_x_Co_3‐x_O_4_ NPs were investigated as an anode electrode for SIBs in an ether‐based electrolyte. Benefiting from the high diffusion contribution (8.64 × 10^−12^ cm^2^ s^−1^), and the good electrical conductivity (11.64 × 10^−3^ S cm^−1^), as‐prepared Fe_x_Co_3‐x_O_4_ NPs endow to achieve a remarking rate capability of 402.9 mAh g^−1^ at 3 A g^−1^ and cycling stability barely without capacity fading 786.2 mAh g^−1^ after 500 cycles at 0.5 A g^−1^. We also used ex situ XRD and in situ GEIS methods to analyze the conversion reaction mechanism of sodium storage and proposed that iron lattice doping can increase the conductivity and decrease electronic resistance during discharge/charge processes of Fe_x_Co_3‐x_O_4_ NPs. The DFT calculation and UV–vis results confirmed that the Fe_x_Co_3‐x_O_4_ NPs electrode is more favorable for decreasing the bandgap (1.57 eV→1.38 eV) with the lattice doping. Iron lattice doping can effectively increase the Co─O bond length, expanding the ion transport channels. In addition, the as‐assembled Na‐ion full cell displays a reversible capacity of 105 mAh g^−1^ and good cycling durability ≈200 cycles. All these effects work together to obtain stable final high‐rate performance. This work provides a promising anode material that may lead to the development of high‐rate conversion materials with stable cycling for prospective energy storage applications.

## 4 Experimental Section

### Materials Synthesis

A co‐precipitation doping synthesis method was designed to prepare Fe‐doped Co_3_O_4_ nanoparticles (Fe_x_Co_3‐x_O_4_ NPs). In a typical process (6%‐Fe_x_Co_3‐x_O_4_ NPs), 25 mm of Co(NO_3_)_2_·6H_2_O, 15 mm of Fe(NO_3_)_3_·9H_2_O, 46 mm of Urea and 68 mm of Ammonium fluoride (NH_4_F) were dissolved in DI water and stirred for 1 h till the solution turned light pink. The solution was transferred into two 50 mL Teflon‐lined autoclaves, respectively, and then they were heated at 120 °C for 12 h. After that, they were cooled down to room temperature. Then, the processor products were collected by a centrifuge machine and washed with DI water and ethanol several times, followed by a vacuum drying process at 100 °C for 12 h. Finally, the processor was obtained by annealing the processor at 400 °C for 2 h under an Air atmosphere. When comparing the other samples (0%, 3%, 9%), all the conditions were kept the same, except for the difference in the content of Fe(NO)_3_.

### Materials Characterization

The crystalline structures of Fe_x_Co_3‐x_O_4_ NPs were identified with the X‐ray diffractometer (XRD, SIEMENS D5000). The samples were scanned from 2θ = 10 ° to 90 ° at a rate of 0.02° s^−1^ in Bragg‐Brentano geometry and a rate of 0.01° s^−1^ in Grazing‐Incidence XRD with a copper (Cu) anode and Kα radiation. The samples were analyzed by XPS using a spectrometer (Thermos Scientific K‐Alpha) with monochromatic Al‐Kα radiation. Raman spectroscopy (WITec) with a He–Ne laser (532 nm) was used to explore the vibration modes of Co─O bonds. The precise and accurate atomic ratio of Fe/Co was identified with the inductively coupled plasma‐optical emission spectrometer (ICP‐OES Agilent 5110). The morphologies and microstructures of samples were observed by an SEM (Hitachi S‐4800) and an HRTEM (JEOL JEM‐435 2100F). The corresponding elemental mapping was also investigated. The Mössbauer measurements were carried out at room temperature in transmission geometry with a custom‐made miniaturized Mössbauer Spectrometer (MIMOS II) in self‐production. A Co‐57/Rh source was used and the measurements were recorded at 14.4 keV. Data analysis was performed using the “Recoil” software (Rancourt and Ping, 2003). The velocity scale and isomer shift δ were calibrated with α‐Fe (iron foil with 2.14% Fe‐57, 20 µm thick, 99.85% purity). Iron species were assigned by comparison of measured Mössbauer parameters to literature data. Resistivity measurements were made with a 4D four‐point probe employing a Keithley 2400 programmable current source.

### Electrochemical Measurements

Working electrodes were fabricated by mixing the nanosheets assemblies, Super P, and carboxymethyl cellulose sodium with a weight ratio of 8:1:1. The mixture was uniformly coated (doctor‐blade) on copper foils with a typical mass loading of around 1 mg cm^−2^. It was then dried at 80 °C under a vacuum for over 12 h. Electrochemical measurements were performed using the configuration of coin cells CR2032 and the cells were assembled in an argon‐filled glove box with oxygen and moisture concentrations below 0.1 ppm. Na disc as the counter electrode was separated from the working electrode by a glass microfiber filter (Whatman, Grade GF/B). Electrolyte was 1 m sodium hexafluorophosphate in a 1, 2‐dimethoxyethane (DME) solution was prepared in a nitrogen‐filled glove box. Galvanostatic charge/discharge was carried out on a Land CT 2001A battery testing system (Land. China) at rates of 0.1–5 A g^−1^ at room temperature. Cyclic voltammetry (CV) and electrochemical impedance spectroscopy (EIS) were carried out by a VSP electrochemical workstation (Bio‐Logic, France). To assemble Na‐ion full cells, the Fe_x_Co_3‐x_O_4_ anode was pre‐sodiation through five galvanostatic cycles at 50 mA g^−1^, which served to offset the initial capacity loss. Following this treatment, the prepared anode was combined with the PB cathode at a mass ratio of 1.2:1. The full cell's specific capacity was determined based on the mass of the PB cathode.

### Calculation Method

First‐principles calculations based on DFT were carried out using the Vienna Ab initio Simulation Package (VASP).^[^ ^63^ ^]^ The PBE + U_eff_ calculations were employed. The onsite Coulomb interaction U_eff_ was 3.5 eV for the Co ions and 4.3 eV for the Fe ions.^[^ ^64^, ^65^ ^]^ The initial magnetic moments were set. where 4 µB for Co^2+^, 0 µB for Co^3+^, 5.9 µB for Fe^3+^, and 0.6 µB for O^2−^ throughout all calculations. A plane‐wave cut‐off energy of 700 eV was applied and the convergence criteria for energy and forces were set to 1 × 10^−8^ eV and 0.02 eV Å^−1^, respectively. A Γ‐centered 4 × 4 × 4 *k*‐point mesh was used for the structural optimization. While the single‐point calculations were performed using an increased 5 × 5 × 5 *k*‐point mesh. Based on the tetrahedron method with Blöchl's corrections.^[^ ^66^ ^]^ The electronic band structures were computed along high‐symmetry points in the Brillouin zone. Specifically, W (0.500, 0.250, 0.750), L (0.500, 0.500, 0.500), Γ (0.000, 0.000, 0.000), X (0.500, 0.000, 0.500), and K (0.375, 0.375, 0.750). The bandgap information was summarized by the VASPkit script.^[^ ^67^ ^]^ Cobalt oxides crystalized in Spinel structure and Abhay reported that the unit cell can be doped with Fe ions at 16*d* Wyck site in trace amount.^[^ ^58^ ^]^ The initial model cells were constructed based on the real model of Co_24‐x_Fe_x_O_32_ (0 ≤ x ≤ 4). with increasing the randomly doping of iron atoms into the unit cell.

## Conflict of Interest

The authors declare no conflict of interest.

## Supporting information



Supporting Information

## Data Availability

The data that support the findings of this study are available in the supplementary material of this article.
