# Supplementary material for: Facilitating Sodium‐Ion Diffusion in Fe‐Doped Co3O4 for High‐Rate Performance
Source: Small. 2025 Feb 28;22(12):2412449. doi: 10.1002/smll.202412449 (PMC12934381; doi:10.1002/smll.202412449)
Supplement: Supplementary file 1 — Supporting Information [file SMLL-22-2412449-s001.docx]

**Electronic supplementary information (ESI)**

**Facilitating Sodium-Ion Diffusion in Fe-Doped Co_3_O_4_ for High-Rate Performance**

Yonghuan Fu^1^, Guowei Sun^2^, Rene Lucka^3^, Qijun Song^2^, Franz Renz^3^, Huaping Zhao^1^, Zhijie Wang^4^ and Yong Lei^1,^ *

^1^Fachgebiet Angewandte Nanophysik, Institut für Physik & IMN MacroNano, Technische Universität Ilmenau, 98693 Ilmenau, Germany
^2^ School of Chemical and Material Engineering, Jiangnan University, 214122 WuXi, China

^3^Institut für Anorganische Chemie, Leibniz Universität Hannover, 30167 Hannover, Germany

^4^ Key Laboratory of Semiconductor Materials Science, Center of Materials Science and Optoelectronics Engineering, University of Chinese Academy of Sciences, 100049 Beijing, China

*Corresponding author E-mail: yong.lei@tu-ilmenau.de

# Supporting Figures

d_(311)_=0.243 nm

d_(311)_=0.275 nm

(a)

(b)

Figure S1. The HRTEM images of (a) Fe_x_Co_3-x_O_4_ and (b) Co_3_O_4_ NPs.

**Figure S2.** The different doping content detailed XRD of Fe_x_Co_3-x_O_4_ electrode.


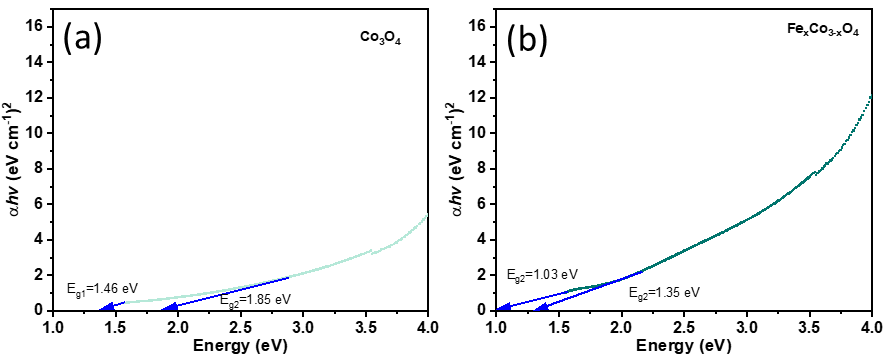


**Figure S3.** Tau plot of UV-visible absorption data for the calculation of band gap energy of the (a) Co_3_O_4_ and (b) Fe_x_Co_3-x_O_4_.

**Figure S4.** XPS spectra of Co 2p of Fe_x_Co_3-x_O_4_ NPs and Co_3_O_4_ NPs.

**Figure S5.** XPS spectra of O 1s of Fe_x_Co_3-x_O_4_ NPs and Co_3_O_4_ NPs.

**Figure S6.** XPS spectra of Fe 2p of Fe_x_Co_3-x_O_4_ NPs and Co_3_O_4_ NPs.


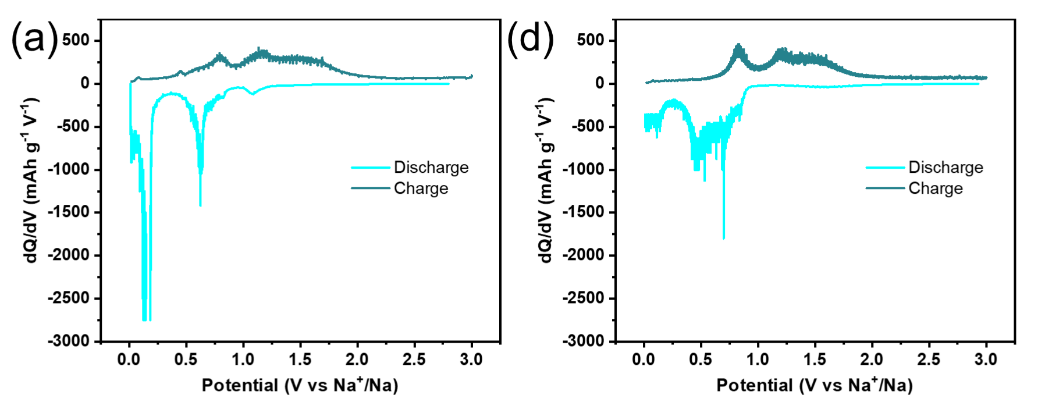


**Figure S7.** Differential capacity *vs.* voltage (dQ/dV) curves for the (a) Fe_x_Co_3-x_O_4_ NPs and (b) Co_3_O_4_ NPs.

**Figure S8.** CV curves of Co_3_O_4_ NPs electrode within 0.01-3 V at a scan rate of 0.1 mV s^−1^.


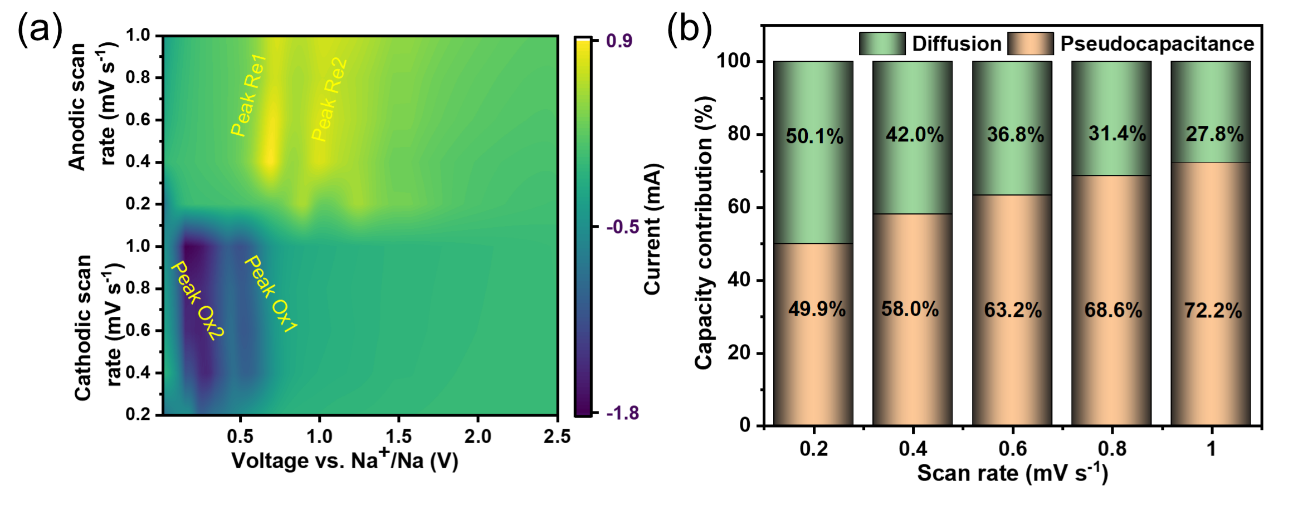


**Figure S9.** (a) The contour of Co_3_O_4_ NPs electrode at different scanning rates; (b) Capacity contributions at different scan rates with the yellow part describing the capacitive contribution and the green area standing for the diffusion-controlled capacity for Co_3_O_4_ electrode.

**Figure S10.** Kinetic analysis of the electrochemical behavior of the Fe_x_Co_3-x_O_4_ electrode versus Na^+^/Na, separation of the capacitive and diffusion currents at a scan rate of 0.6 mV s^−1^.


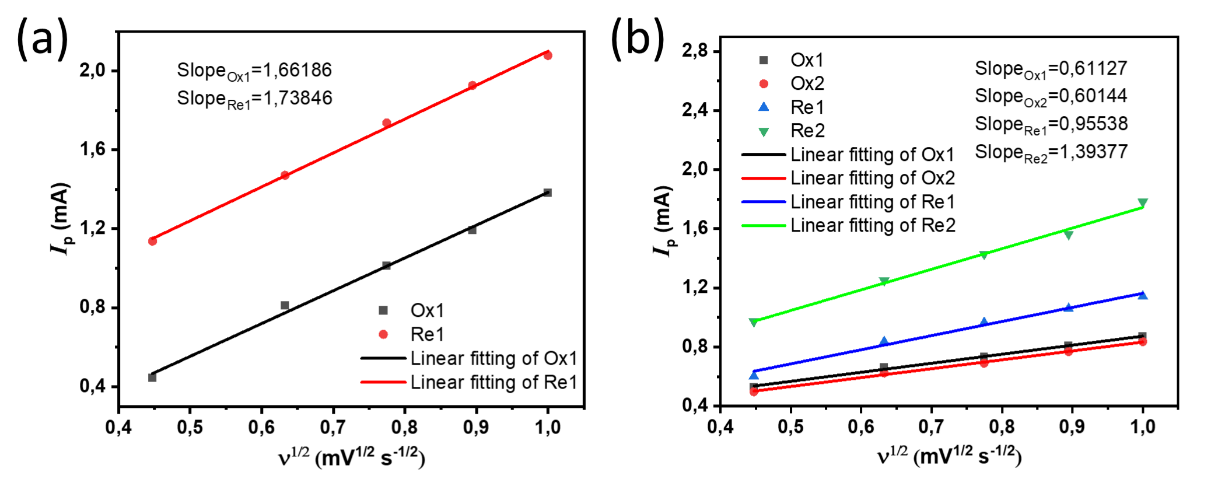


**Figure S11.** Liner fitted b-value according to the relationship of the log (scan rate) and log (peak current) for (a) Fe_x_Co_3-x_O_4_ and (b) Co_3_O_4_ electrode.


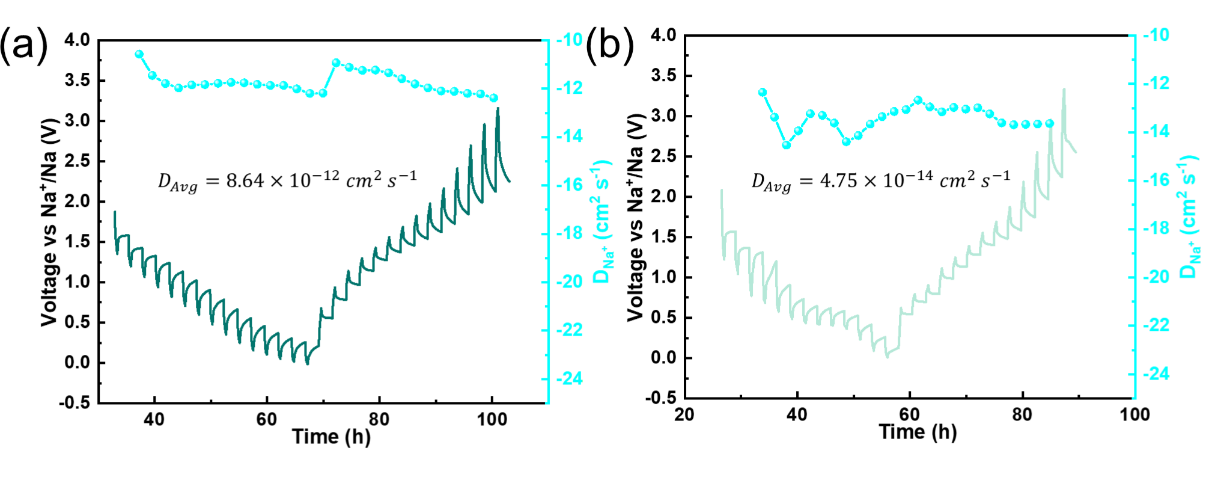


**Figure S12.** Comparison of GITT results (a) Fe_x_Co_3-x_O_4_ electrode; (b) Co_3_O_4_ electrode.


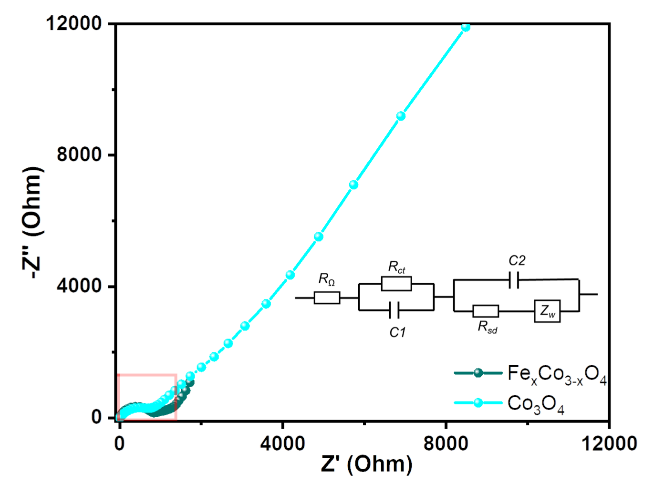


**Figure S13.** Nyquist plots of Fe_x_Co_3-x_O_4_ and Co_3_O_4_ electrode.

The Nyquist plots of the Fe_x_Co_3-x_O_4_ NPs electrodes at different voltage were recorded in the frequency range of 100 kHz to 100 mHz. The impedance spectra are fitted using the equivalent circuit model. and the fitted impedance parameters are listed in **Table S6**. The equivalent circuit model contains electrolyte resistance and SEI resistance (R_Ω_). charge transfer resistance (R_ct_). solid state diffusion in electrode (R_sd_). Warburg impedance(Z_w_) and two constant phase elements (C1 and C2).


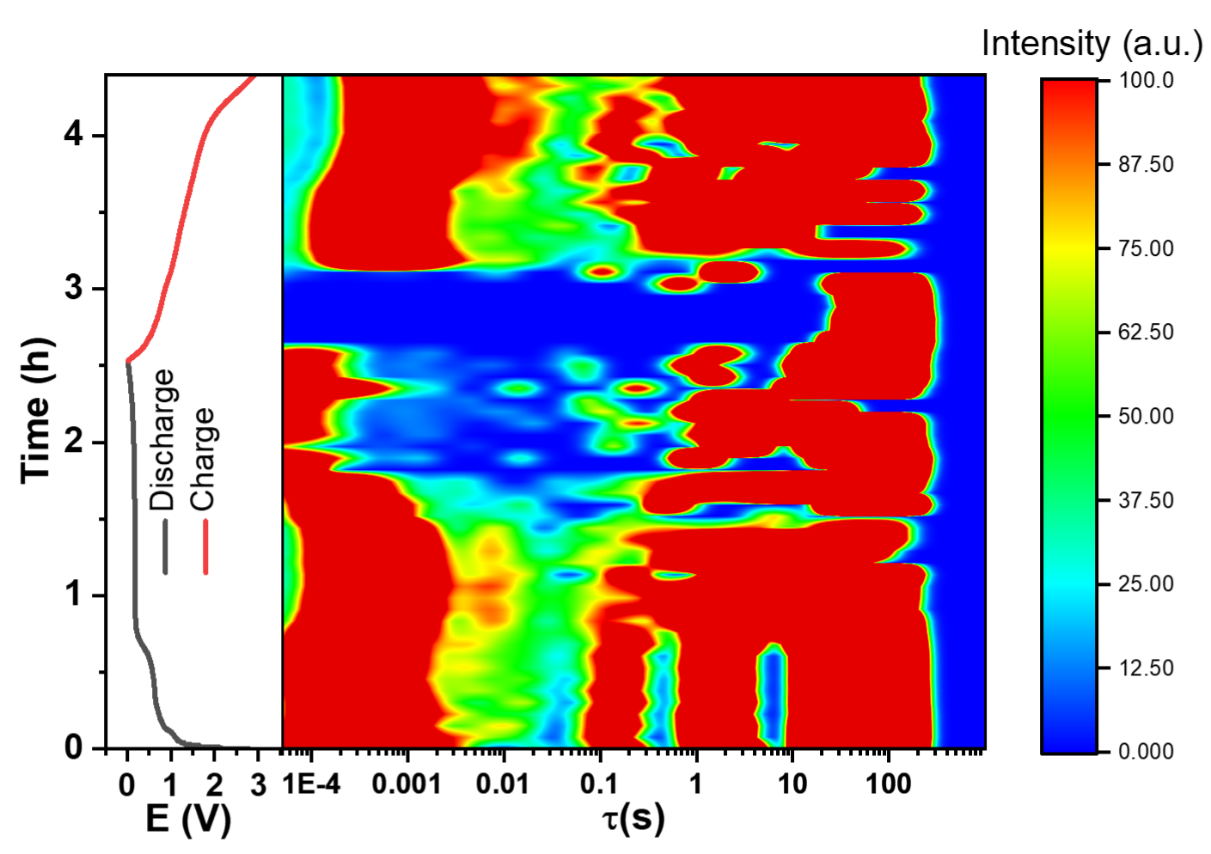


**Figure S14.** Distribution of relaxation time (DRT) analysis based on the in situ-GEIS of Co_3_O_4_ NPs sample half battery.


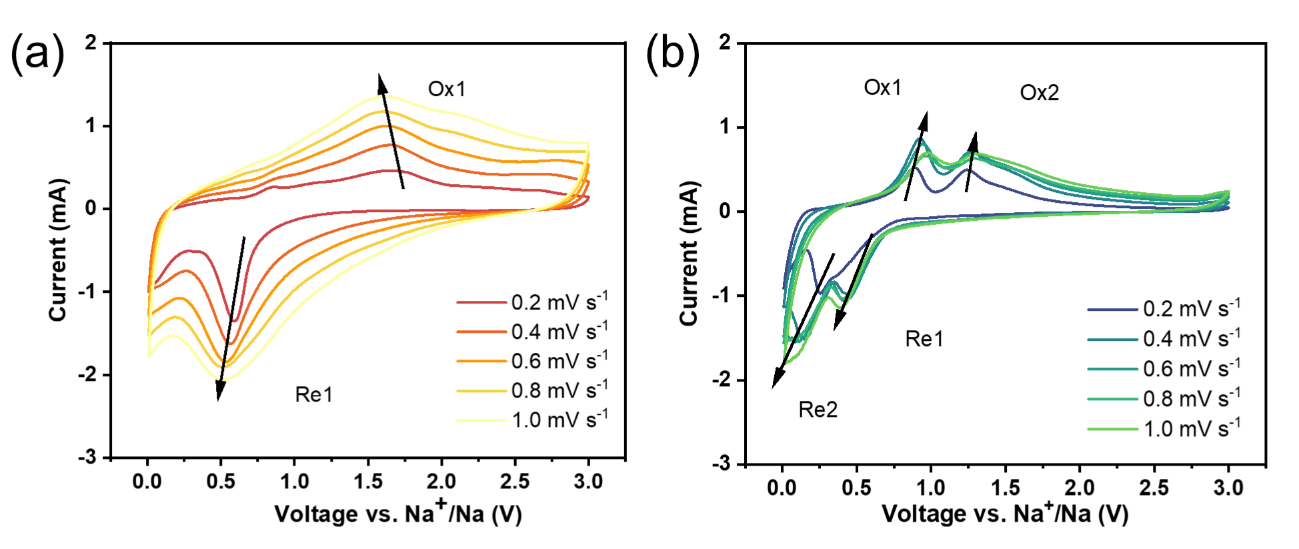


**Figure S15.** CV curves at different scanning rates (a) Fe_x_Co_3-x_O_4_ (b) Co_3_O_4_.


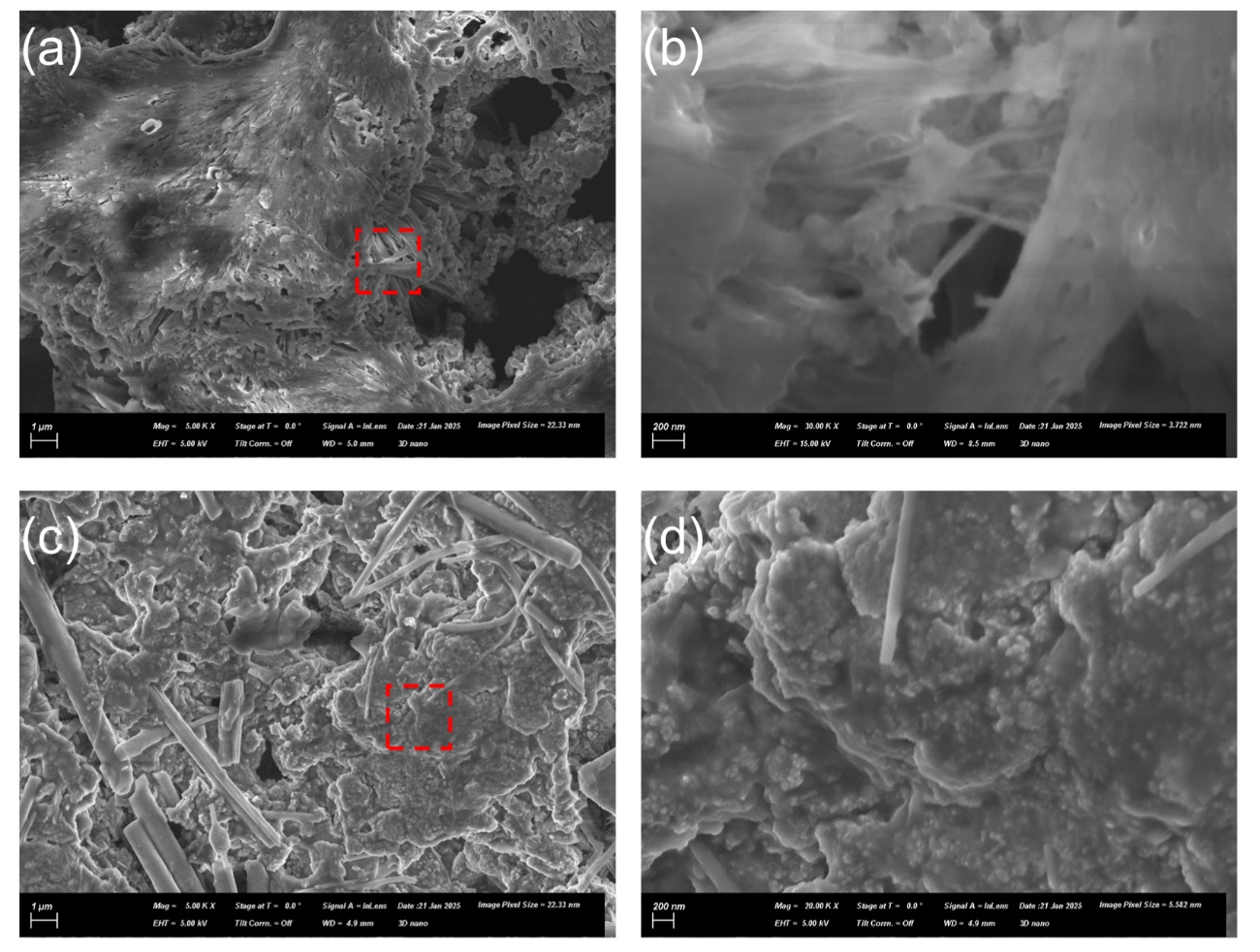


**Figure S16.** The FESEM image of the after 200 cycles samples. (a, b) Fe_x_Co_3-x_O_4_, (c, d) Co_3_O_4_.


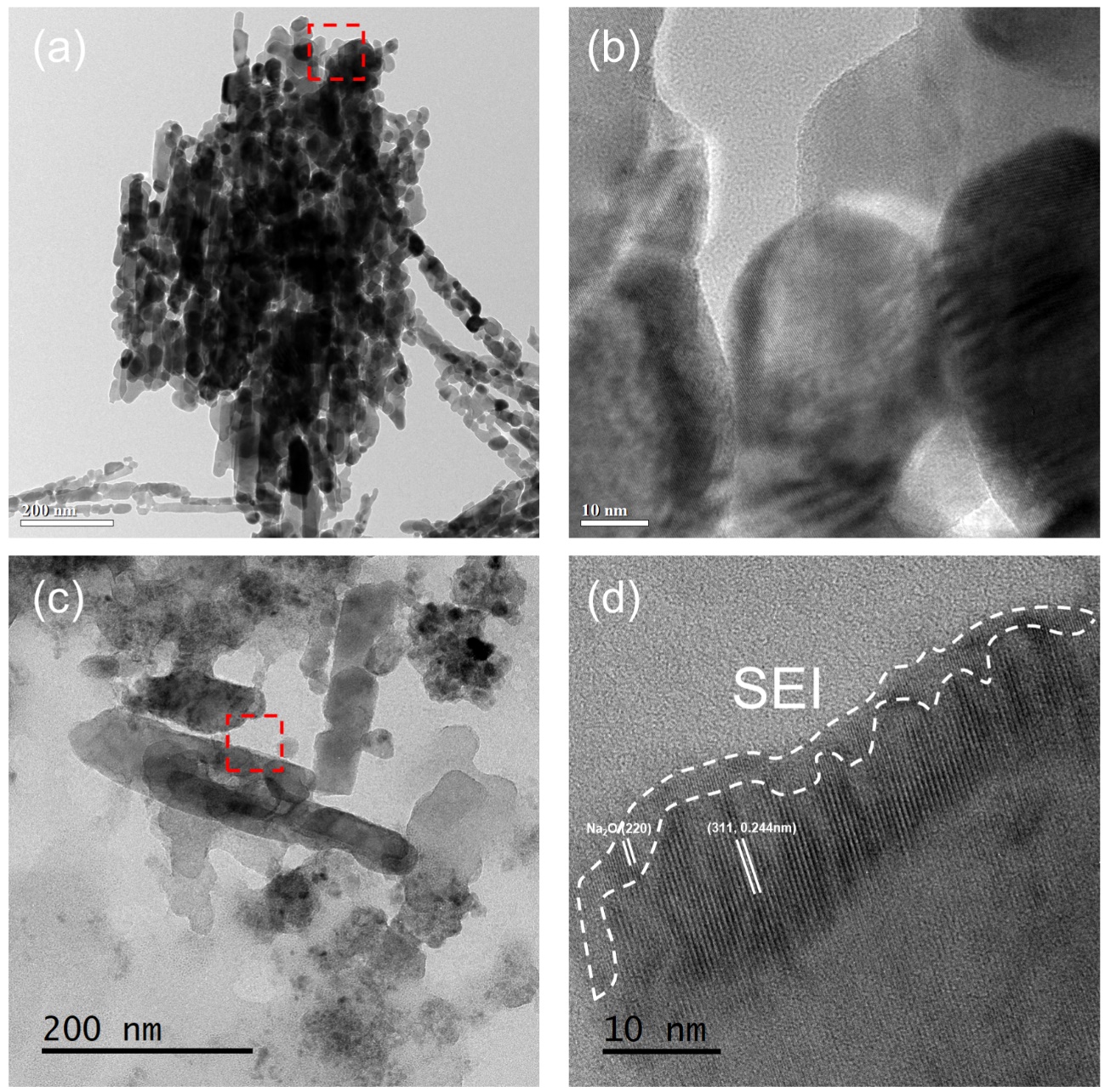


**Figure S17.** TEM micrographs of Fe_x_Co_3-x_O_4_ NPs (a, b) before and (c, d) after 200 cycles.


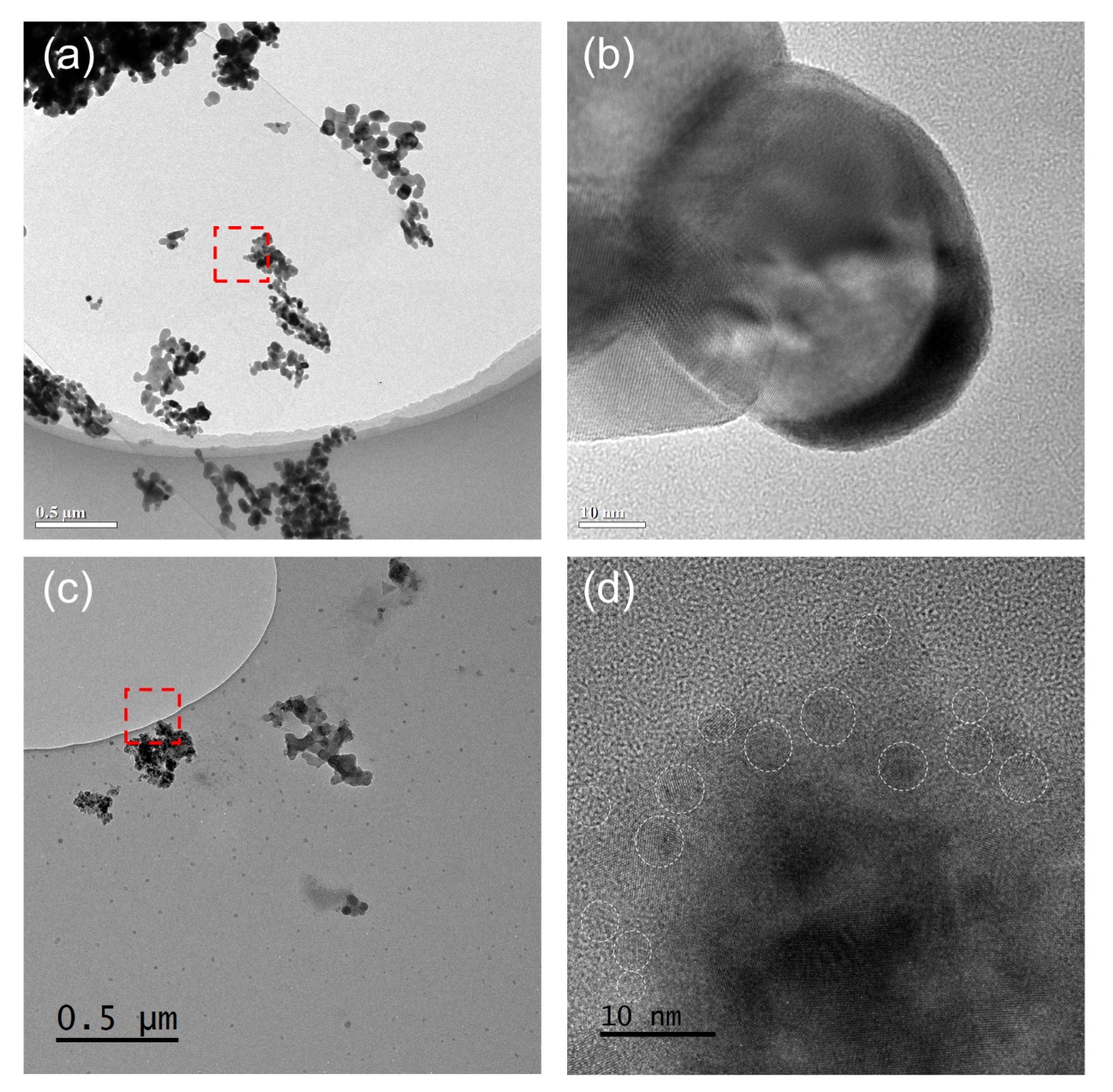


**Figure S18.** TEM micrographs of Co_3_O_4_ NPs (a, b) before and (c, d) after 200 cycles.


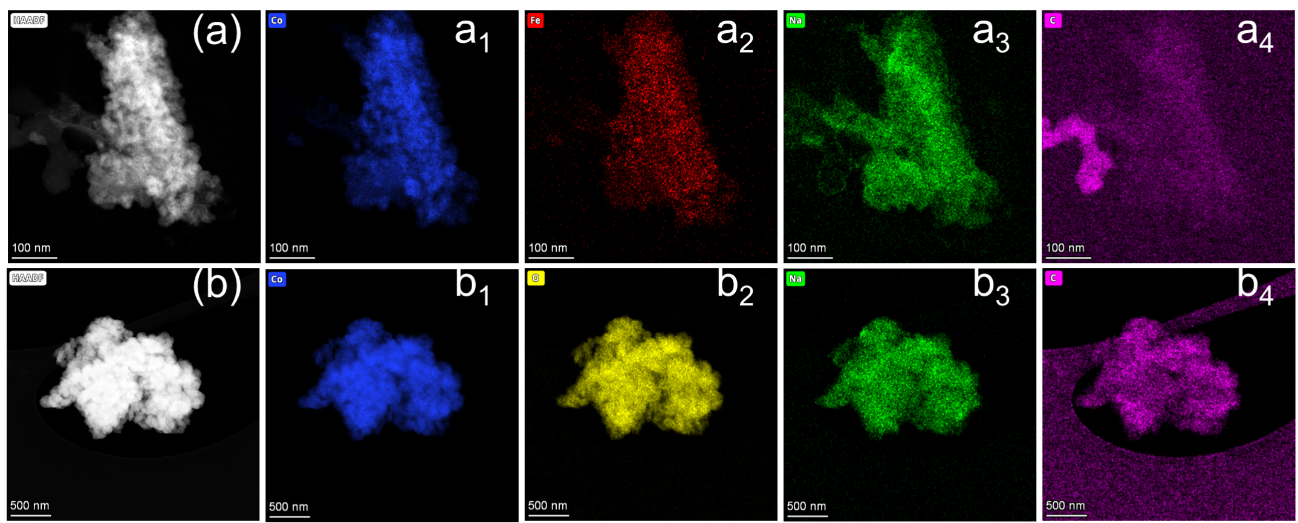


**Figure S19.** Elemental mapping images of the electrode after 200 cycles, (a) Fe_x_Co_3-x_O_4_ NPs electrode and (b) Co_3_O_4_ NPs electrode.


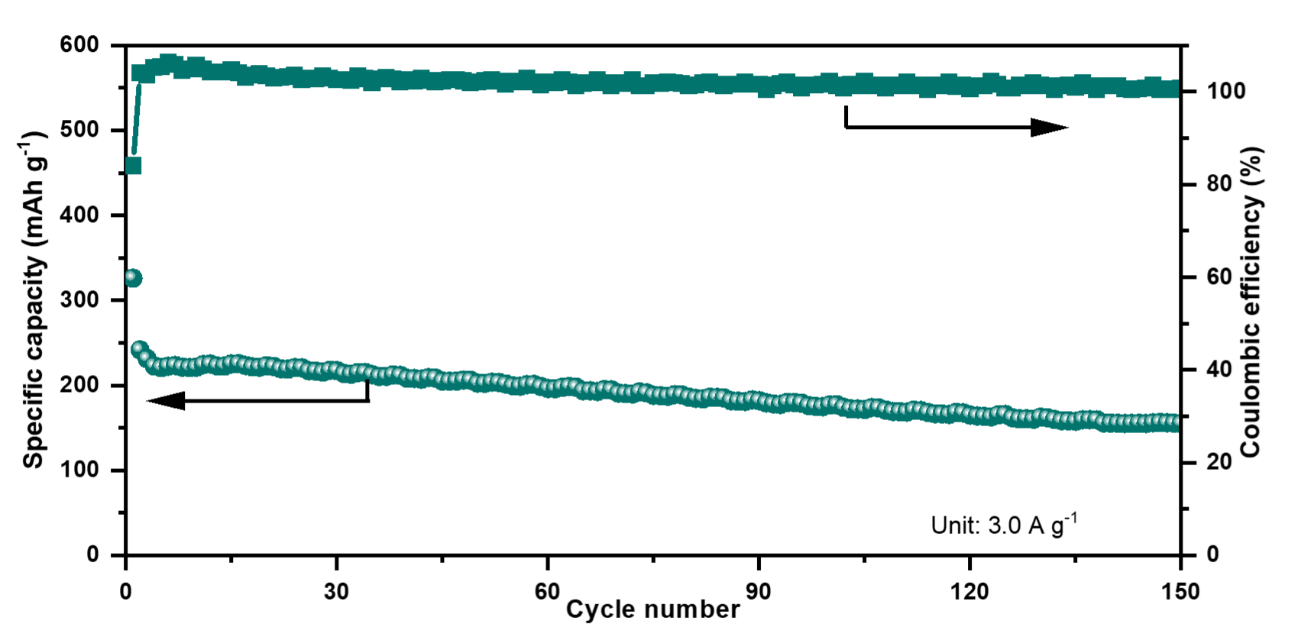


**Figure S20.** Cycling performance of Fe_x_Co_3-x_O_4_ NPs electrode at 3.0 A g^−1^ within 0.01-3 V.


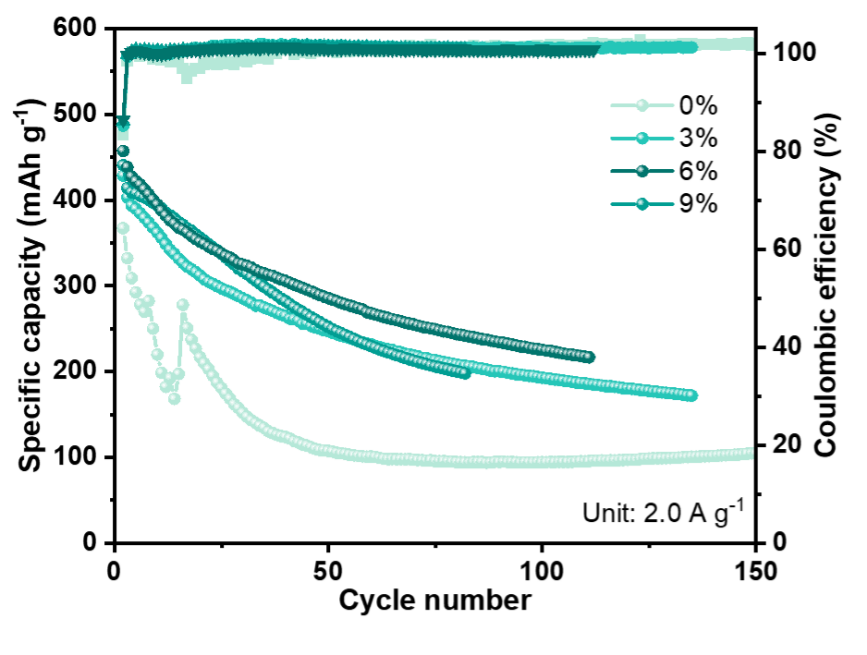


**Figure S21.** Cycling performance of different Fe doped content at 2.0 A g^−1^ within 0.01-3 V.

# Supporting Formula

Tauc relation from Equation 1:

$\left( \alpha hv \right)^{n}=A(hv-E_{g})$ (1)

Where, *hv* is photon energy. A is constant. *E_g_* is the bandgap energy α is absorption coefficient and *n* is the constant.

Studies have shown that there is an exponential function between the current response and the corresponding sweep rate. and the following equations are introduced to elucidate the relationship. Where *i* is the peak current. *v* is the scan rate. and *a* and *b* are adjustable parameters. The *b* value can represent the type of Na^+^ insertion/extraction and can be determined by plotting log(*i*) against log(*ν*). where *K_1_* and *K_2_* are constants.

𝑖 = 𝑎𝑣^𝑏^  (2)

𝑙𝑜𝑔(𝑖) = 𝑏𝑙𝑜𝑔(𝑣) + log (𝑎) (3)

𝑖(𝑉) = 𝐾_1_𝑣 + 𝐾_2_𝑣^1/2^ (4)

I_p_ = (2.69 × 10^5^) n^3/2^SD^1/2^C_0_*v*^1/2^ (5)

The Na^+^ ions diffusion coefficient (D_GITT_) was calculated based on Equation 6:

$D_{GITT}=\frac{4}{\pi\tau}{(\frac{m_{B}V_{M}VM}{M_{B}A})}^{2}{(\frac{\Delta E_{s}}{\Delta E_{r}})}^{2}$ (6)

The lattice distortion is calculated based on Equation 7:

${(2\beta)}^{2}{\cos\theta}^{2}=\frac{4}{\pi^{2}}{(\frac{\lambda}{D_{hkl}})}^{2}+32<{\varepsilon_{hkl}}^{2}>{\sin\theta}^{2}$ (7)

The calculation of lattice parameters variation during in situ XRD was based on the Bragg equation and Equation 8:

$d_{hkl}=\frac{1}{\sqrt{\frac{4}{3}\times\frac{(h^{2}+hk+k^{2})}{a^{2}}+{(\frac{l}{c})}^{2}}}$ (8)

# Supporting Tables

**Table S1.** The electrical conductivity. band gaps. BET surface area and pore radius of Fe_x_Co_3-x_O_4_ with different doping contents.

| **Samples** | **Electrical conductivity ×10^-3^ S cm^-1^** | **Band gap**  **(eV)** | | **BET surface area (m^2^ g^-1^)** | **Pore radius (nm)** |
| --- | --- | --- | --- | --- | --- |
|  |  | **E_g1_** | **E_g2_** |  |  |
| 0 %-Fe_x_Co_3-x_O_4_ | 4.32 | 1.46 | 1.85 | 5.70 | 8.82 |
| 3 %-Fe_x_Co_3-x_O_4_ | 6.57 | 1.15 | 1.50 | 11.52 | 10.46 |
| 6 %-Fe_x_Co_3-x_O_4_ | 11.64 | 1.05 | 1.40 | 20.99 | 15.32 |
| 9 %-Fe_x_Co_3-x_O_4_ | 8.28 | 1.03 | 1.35 | 15.65 | 13.45 |

**Table S2.** Cell parameters. grain size and lattice distortion of Fe_x_Co_3-x_O_4_ with different doping levels obtained from XRD refinement data.

| **Materials** | **Lattice Parameters (nm)** | | | | | | **D_(311)_ (nm)** | **˂ε_hkl_^2^˃^1/2^**  **× 10^2^** |
| --- | --- | --- | --- | --- | --- | --- | --- | --- |
|  | a | b | c | α | β | γ |  |  |
| 0 %-Fe_x_Co_3-x_O_4_ | 0.805 | 0.805 | 0.805 | 90 | 90 | 90 | 13.298 | 3.394 |
| 3 %-Fe_x_Co_3-x_O_4_ | 0.807 | 0.807 | 0.807 | 90 | 90 | 90 | 19.256 | 2.541 |
| 6 %-Fe_x_Co_3-x_O_4_ | 0.809 | 0.809 | 0.809 | 90 | 90 | 90 | 11.485 | 2.183 |
| 9 %-Fe_x_Co_3-x_O_4_ | 0.812 | 0.812 | 0.812 | 90 | 90 | 90 | 13.411 | 2.761 |

**Table S3.** The elemental content of 6%-Fe_x_Co_3-x_O_4_ NPs.

| **Element** | **Theo. At%** | **Meas. At%** |
| --- | --- | --- |
| Co | 67.68 | 67.11 |
| O | 26.49 | 27.24 |
| Fe | 5.80 | 5.65 |

According to the ICP test data the corresponding atomic ratios can be obtained to obtain the chemical formula of Fe_0.25_Co_2.75_O_3.964_.

**Table S4.** ^57^Fe Mössbauer parameters obtained from the analysis of the Mössbauer spectra results.

| Samples | Species | $\delta\left( \frac{\mathrm{mm}}{s} \right)$ | $\Delta E_{Q} \left( \frac{\mathrm{mm}}{s} \right)$ | $H \left( T \right)$ | Area (%) |
| --- | --- | --- | --- | --- | --- |
| 5 %-Fe_x_Co_3-x_O_4_ | Fe(III, high) | 0.367 | -0.022 | 37.316 | 72.446 |
|  | Fe(III, low) | 0.392 | 0.026 | 30.472 | 27.553 |
| 7 %-Fe_x_Co_3-x_O_4_ | Fe(III, high) | 0.353 | -0.030 | 37.578 | 68.994 |
|  | Fe(III, low) | 0.353 | 0.035 | 33.947 | 31.006 |
| 10 %-Fe_x_Co_3-x_O_4_ | Fe(III, high) | 0.357 | -0.024 | 37.832 | 67.029 |
|  | Fe(III, low) | 0.304 | -0.083 | 33.263 | 32.970 |
| 15 %-Fe_x_Co_3-x_O_4_ | Fe(III, high) | 0.370 | -0.023 | 37.079 | 69.790 |
|  | Fe(III, low) | 0.329 | -0.028 | 32.336 | 30.209 |

Since the minimum iron doping content in the Mössbauer is 5%, four samples with different iron doping contents were synthesized for testing based on the same approach.

**Table S5.** The kinetic control situations of Co_3_O_4_ NPs and Fe_x_Co_3-x_O_4_ NPs.

| Scan rate (mV/s) | Co_3_O_4_ NPs | | Fe_x_Co_3-x_O_4_ NPs | |
| --- | --- | --- | --- | --- |
|  | Diffusion | Pseudocapacitance | Diffusion | Pseudocapacitance |
| 0.2 | 50.11 % | 49.89 % | 76.32 % | 23.68 % |
| 0.4 | 42.02 % | 57.98 % | 68.35 % | 31.65 % |
| 0.6 | 36.83 % | 63.17 % | 59.22 % | 40.78 % |
| 0.8 | 31.44 % | 68.56 % | 51.31 % | 48.69 % |
| 1.0 | 27.83 % | 72.17 % | 45.33 % | 54.67 % |

**Table S6.** Fitting results of resistances in the equivalent circuit.

| Potential (V) | | R_sd_ (Ω) | R_ct_ (Ω) | R_Ω_(Ω) |
| --- | --- | --- | --- | --- |
| Discharge | OCV | 332.786 | 21.45 | 3.905 |
|  | 1.80 | 289.99 | 21.838 | 2.332 |
|  | 0.60 | 67.05 | 513.821 | 2.561 |
|  | 0.20 | 203.556 | 0.003 | 11.887 |
| Charge | 0.80 | 92.054 | 0.001 | 0.184 |
|  | 1.20 | 29.838 | 5.528 | 219.169 |
|  | 2.10 | 264.753 | 0.004 | 0.006 |

Theoretical density functional theory (DFT) calculations were conducted to evaluate the impact of iron lattice doping on the electrical conductivity of Co_3_O_4_. In this study, we designed a series of calculations by varying the iron components within the Co_24_O_32_ unit cell. By introducing different numbers of iron atoms, we established five stoichiometric models comprising Co_24_O_32_, Fe_1_Co_23_O_32_, Fe_2_Co_22_O_32_, Fe_3_Co_21_O_32_, Fe_4_Co_20_O_32_, corresponding to doping concentrations of 0%, 3%, 6%, 9%, and 11%, respectively. The important theoretical calculation parameters are listed in Table S7.

**Table S7.** Simulated lattice parameters. bond lengths. and band gaps of pristine Co_3_O_4_ and its variants with doped iron atoms.

| Stimulation Data | Co_24_O_32_  (0%) | Fe_1_Co_23_O_32_  (3%) | Fe_2_Co_22_O_32_  (6%) | Fe_3_Co_21_O_32_  (9%) | Fe_4_Co_20_O_32_  (11%) |
| --- | --- | --- | --- | --- | --- |
| Cell parameter:  a (Å) | 8.05 | 8.09 | 8.12 | 8.13 | 8.15 |
| Co 8*a*- O(Å) | 1.93 | 1.92 | 1.90 | Highly dispersed | Highly dispersed |
| Co 16*d* – O(Å) | 1.88 | 1.93 | 1.93 | Highly dispersed | Highly dispersed |
| Fe 16*d* – O(Å) | - | 2.00 | 2.03 | 1.97 ~ 2.00 | 2.01~2.03 |
| Band gap(eV) | 1.57 | 1.39 | 1.38 | 1.36 | 1.29 |
| Spin up(eV) | 1.75 | 1.60 | 1.58 | 1.58 | 1.53 |
| Spin down(eV) | 1.57 | 1.39 | 1.47 | 1.50 | 1.49 |

**Table S8**. Summary of cobalt oxide as anode electrode reported recently and their corresponding electrochemical performance.

| **Materials** | **Electrolytes**  **(solvents. v/v)** | **Capacity**  **(mA h g ^1^)** | **Rate Capacity**  **(mA h g^‑1^)** | **ICE**  **(%)** | **Capacity retention** | **Voltage**  **window**  **(V)** | **Ref.** |
| --- | --- | --- | --- | --- | --- | --- | --- |
| Our work | 1M NaPF_6_  (2% FEC in EC: DEC=1: 1) | 875 at 0.5A g^-1^ | 243 at 5.0 A g^-1^ | 85 | -88% after 500 cycles (0.5 A g^-1^) | 0.01-3.0 | - |
| Co_3_O_4_ | 1M NaClO_4_  (propylene carbonate/PC and 2% fluoroethylene carbonate/FEC) | 220 at 0.025 A g^-1^ | - | 43 | -86% after 50 cycles (0.05 A g^-1^) | 0.01-3.0 | ^[1]^ |
| Co_3_O_4_ nanoparticles | 1M NaClO_4_  (PC : 2% FEC =95: 5) | 712 at 0.1 A g^-1^ | - | 65 | -75% after 500 cycles (0.5 A g^-1^) | 0.01-3.0 | ^[2]^ |
| 2D assembly of Co_3_O_4_ nanoparticles (CAN) | 1M NaClO_4_  (PC and 2% FEC) | 566 at 0.1 A g^-1^ | 175 at 1.6 A g^-1^ | 54 | -70% after 100 cycles (0.5 A g^-1^) | 0.01-3.0 | ^[3]^ |
| Co_3_O_4_@CNTs | 1M NaPF_6_  (ethylene carbonate/EC: diethyl carbonate/DEC=1: 1) | 487 at 0.1 A g^-1^ | 184 at 3.2 A g^-1^ | 64 | -90% after 30 cycles (0.3 A g^-1^)- | 0.01-3.0 | ^[4]^ |
| Co_3_O_4_/MCNTs | 1M NaPF_6_  (EC: PC=1: 1) | 293 at 0.035 A g^-1^ | - | 52 | -72% after 100 cycles (0.1 A g^-1^) | 0.01-2.5 | ^[5]^ |
| Co_3_O_4_ nanocubes/CNTs | 1M NaClO_4_  (EC: DEC: FEC=1: 1: 0.05) | 495 at 0.1 A g^-1^ | 110 at 1.6 A g^-1^ | 71 | -54% after 100 cycles (1 A g^-1^) | 0.01-3.0 | ^[6]^ |
| Shale-like Co_3_O_4_ | 1M NaClO_4_  (EC: PC=1: 1) | 327.3 at 0.05 A g^-1^ | 153.8 at 5.0 A g^-1^ | 59 | -101% after 50 cycles (5 A g^-1^) | 0.005-2.9 | ^[7]^ |
| Co_3_O_4_ MNSs/3DGNs | 1M NaClO_4_  (propylene carbonate/PC and 2% fluoroethylene carbonate/FEC) | 375 at 0.025 A g^-1^ | 82.3 at 0.5 A g^-1^ | 56 | -123% after 50 cycles (0.1 A g^-1^) | 0.01-3.0 | ^[8]^ |
| Hollow Co_3_O_4_ microspheres | 1M NaClO_4_  (PC) | 757 at 0.035 A g^-1^ | 650 at 0.89 A g^-1^ | 45 | -38% after 10 cycles (1 A g^-1^) | 0.01-2.0 | ^[9]^ |
| Co_3_O_4_@C | 1M NaClO_4_  (PC and 5 % FEC) | 416 at 0.2 A g^-1^ | - | 53 | -55% after 150 cycles (1 A g^-1^) | 0.01-3.0 | ^[10]^ |
| Co_3_O_4_@NC | 1M NaClO_4_  (PC and 2 % FEC) | 516 at 0.1 A g^-1^ | 263 at 1.0 A g^-1^ | 63 | -87% after 60 cycles (0.1 A g^-1^) | 0.01-3.0 | ^[11]^ |
| Co_3_O_4_-graphene | 1M NaPF6  (diethylene glycol dimethyl ether/DEGDME) | 756 at 0.1 A g^-1^ | - | 85 | -59% after 50 cycles (0.1 A g^-1^) | 0.01-2.5 | ^[12]^ |
| meso-porous Co_3_O_4_ | 1M NaPF6  (5% FEC in EC: DEC=1: 1) | 447 at 0.1 A g^-1^ | 204 at 0.445 A g^-1^ | 64 | -75% after 200 cycles (0.1 A g^-1^) | 0.01-3.0 | ^[13]^ |

# References

1. M. M. Rahman, A. M. Glushenkov, T. Ramireddy, Y. Chen, *Chem. Commun.* **2014**, 50, 5057-5060.

2. M. Xu, Q. T. Xia, J. L. Yue, X. H. Zhu, Q. B. Guo, J. W. Zhu, H. Xia, *Adv. Funct. Mater.* **2019**, 29, 1807377.

3. D. H. Chen, L. L. Peng, Y. F. Yuan, Y. Zhu, Z. W. Fang, C. S. Yan, G. Chen, R. Shahbazian-Yassar, J. Lu, K. Amine, G. H. Yu, *Nano Lett.* **2017**, 17, 3907-3913.

4. Z. L. Jian, P. Liu, F. J. Li, M. W. Chen, H. S. Zhou, *J. Mater. Chem. A* **2014**, 2, 13805-13809.

5. Q. J. Deng, L. P. Wang, J. Z. Li, *J. Mater. Sci.* **2015**, 50, 4142-4148.

6. Z. Y. Wang, S. G. Zhang, L. C. Yue, B. Wu, J. Mi, *Solid State Ion.* **2017**, 312, 32-37.

7. H. H. Li, Z. Y. Li, X. L. Wu, L. L. Zhang, C. Y. Fan, H. F. Wang, X. Y. Li, K. Wang, H. Z. Sun, J. P. Zhang, *J. Mater. Chem. A* **2016**, 4, 8242-8248.

8. Y. G. Liu, Z. Y. Cheng, H. Y. Sun, H. Arandiyan, J. P. Li, M. Ahmad, *J. Power Sources* **2015**, 273, 878-884.

9. J. W. Wen, D. W. Zhang, Y. Zang, X. Sun, B. Cheng, C. X. Ding, Y. Yu, C. H. Chen, *Electrochim. Acta* **2014**, 132, 193-199.

10. Y. Lu, E. Fong, *Mater. Today Energy* **2017**, 4, 89-96.

11. Y. Wang, C. Y. Wang, Y. J. Wang, H. K. Liu, Z. G. Huang, *J. Mater. Chem. A* **2016**, 4, 5428-5435.

12. H. Kim, H. Kim, H. Kim, J. Kim, G. Yoon, K. Lim, W. S. Yoon, K. Kang, *Adv. Funct. Mater.* **2016**, 26, 5042-5050.

13. K. C. Klavetter, S. Garcia, N. Dahal, J. L. Snider, J. P. de Souza, T. H. Cell, M. A. Cassara, A. Heller, S. M. Humphrey, C. B. Mullins, *J. Mater. Chem. A* **2014**, 2, 14209-14221.
